# Cold-Start Traffic State Forecasting at Unseen Sensor Locations via Support-Conditioned Meta-Graph Learning

**DOI:** 10.3390/s26154995

**Published:** 2026-08-06

**Authors:** Can Wang, Zhiyu Wang, Weijie Wang, Jing Gan, Yanni Ju

**Affiliations:** 1 School of Transportation, Southeast University, Nanjing 211189, China; canwang@seu.edu.cn (C.W.); wang_zhiyu@seu.edu.cn (Z.W.); 230259410@seu.edu.cn (W.W.); 2 School of Modern Posts, Nanjing University of Posts and Telecommunications, Nanjing 210003, China; jinggan@njupt.edu.cn; 3 Department of Road Traffic Management, Sichuan Police College, Luzhou 646000, China; 4 Intelligent Policing Key Laboratory of Sichuan Province, Sichuan Police College, Luzhou 646000, China

**Keywords:** traffic state forecasting, unseen sensor locations, cold-start adaptation, meta-learning

## Abstract

Traffic sensors provide real-time measurements of current traffic conditions, whereas traffic management applications require forecasts of future traffic speed or flow. This study considers the incremental expansion of an operating sensor network, in which a pre-trained network-level forecasting model must predict traffic states at sensor locations that were absent during training. Many spatio-temporal graph neural networks rely on sensor-specific embeddings and graph connections learned from data-rich training networks. These representations are undefined for previously unseen locations, limiting the direct application of pre-trained adaptive-graph forecasters during sensor-network expansion. To address this cold-start problem, we propose support-conditioned sensor-adaptive meta-graph learning (SC-SAMG), which derives target-node representations and spatial dependencies from a short support period. The framework combines a support-set encoder, a task-specific graph learner, and first-order meta-learning to adapt the network-level forecaster using one to seven days of target observations. Experiments on four traffic benchmarks evaluate forecasts of speed or flow over the next 15–60 min under a leakage-controlled held-out-node protocol. SC-SAMG consistently outperforms fine-tuned gated recurrent unit (GRU), adaptive graph convolutional recurrent network (AGCRN), and diffusion convolutional recurrent neural network (DCRNN) baselines. It reduces mean absolute error (MAE) by up to 11% relative to the adaptive-graph baseline and by up to 7% relative to the diffusion convolutional baseline. These results demonstrate the potential of support-conditioned graph adaptation for incorporating previously unseen sensor locations into existing network-level traffic forecasting systems.

## 1. Introduction

Intelligent transportation systems (ITS) increasingly rely on dense networks of roadside and in-road sensors—loop detectors, microwave radars, and connected-vehicle probes—to continuously observe traffic conditions across large road networks [1]. Accurate short-term traffic state forecasting predicts speed, flow, or occupancy from these network-wide observations over horizons ranging from several minutes to approximately one hour [2]. It is a cornerstone capability that enables proactive signal control, congestion mitigation, and traveller information services [3]. Because the operational value of these services degrades quickly when predictions are stale or inaccurate, forecasting models are expected to deliver reliable multi-step estimates directly from continuous sensor measurements. Traffic sensors measure current traffic conditions, whereas the forecasting model uses historical observations from the sensor network to jointly predict future traffic speed or flow. Each sensor is represented as a node in a shared network-level forecaster rather than as an independent predictor.

Over the past five years, spatio-temporal graph neural networks (STGNNs) became one of the most widely adopted paradigms for this task. By representing each sensor as a node and encoding inter-sensor dependencies through a graph, models such as diffusion- and convolution-based recurrent architectures jointly capture temporal dynamics and spatial correlations, substantially outperforming classical statistical and sequence models [4,5,6]. More recent adaptive-graph methods learn the dependency structure directly from data through node embeddings, removing the need for a predefined adjacency matrix [7,8]. The accuracy of these methods, however, rests on a strong and often overlooked assumption: every sensor in the target network already has weeks or months of historical observations. These observations are needed to learn stable sensor-specific node embeddings and a dependency graph describing which sensors help predict one another. Classical macroscopic traffic-flow models, such as fluid, LWR, and cell-transmission formulations, provide physically interpretable descriptions of congestion propagation when boundary inflows, outflows, road capacities, and calibrated fundamental diagrams are available. These models are especially useful for corridor-level simulation and control, but they require network geometry and flow-conservation information that are not fully available in many detector-only forecasting benchmarks. The present work therefore addresses a complementary setting: learning short-term forecasts for sensors with very limited target-domain observations, where the available information is primarily the recent sensor time series rather than a complete physical traffic-flow model.

This data-rich assumption can be violated when an operating sensor network is expanded to newly instrumented corridors or previously unmonitored districts [1,9,10]. Existing sensors continue to provide network-wide observations, whereas the added sensor locations have only a short observation history. Training a spatio-temporal graph neural network from scratch with such limited data is prone to overfitting. This study therefore focuses on locations absent during model training, rather than the replacement of a device at an already modeled location.

The shared forecasting weights of a pre-trained network-level model can generally be reused for sensor locations unseen during training. However, direct reuse does not define the sensor-identity embeddings and graph connections that many adaptive-graph models learn specifically for the training node set [11,12]. It may also fail to account for the demand patterns and spatial relationships of a new location. Ordinary fine-tuning can adapt these components, but with only one to seven days of support data, its performance may be sensitive to the learning rate and stopping point [13,14,15]. A mechanism is therefore needed to derive new-node representations and spatial dependencies from limited support data while efficiently adapting the shared network-level forecaster.

To bridge this gap, we formulate cold-start traffic state forecasting at sensor locations unseen during training as a few-shot spatio-temporal graph learning problem and propose support-conditioned sensor-adaptive meta-graph learning (SC-SAMG). A support-set encoder summarizes limited target observations, from which a graph learner generates target-node representations and spatial dependencies. First-order meta-learning then adapts the shared parameters of the forecaster, support encoder, and graph learner in a few gradient steps. The main contributions are:

- We formulate forecasting future traffic speed or flow at unseen sensor locations as a few-shot spatio-temporal graph learning problem.
- We propose a support-conditioned graph learner that derives target-node representations and spatial dependencies from limited observations instead of fixed sensor-identity embeddings.
- We combine the graph learner with first-order meta-learning to adapt the shared parameters of the forecaster, support encoder, and graph learner, enabling unseen sensor locations to be incorporated into an existing forecasting system.
- We evaluate the framework across different support sizes, adaptation steps, unseen regions, sensor outages, and component ablations.

The remainder of this paper is organized as follows. Section 2 reviews related work on traffic forecasting, graph learning, transfer learning, and meta-learning. Section 3 defines the problem, Section 4 presents the methodology, Section 5 reports experiments with integrated analysis, and Section 6 concludes the paper.

## 2. Related Work

### 2.1. Short-Term Traffic State Forecasting

Short-term traffic forecasting encompasses classical traffic-flow and statistical models as well as deep spatio-temporal architectures. Macroscopic traffic-flow models describe traffic as a conserved flow over road links and estimate congestion propagation from quantities such as inflow, outflow, link capacity, and calibrated speed–density or flow–density relationships. While these models offer strong physical interpretability, applying them to cold-start traffic state forecasting at previously unseen sensor locations requires network-specific flow inputs, road-capacity information, and calibrated fundamental diagrams or other model parameters. Obtaining these inputs and parameters reliably from only a few days of target observations can be challenging [16,17,18]. Recent long-sequence forecasting research has also introduced state-space models with multi-scale adversarial learning to capture traffic-flow variations at different temporal scales while avoiding the quadratic computational cost of Transformer-based architectures [19]. Early approaches such as autoregressive integrated moving average and Kalman filtering capture linear temporal regularities but struggle with the nonlinearity and abrupt transitions of real traffic [20]. Recurrent, temporal-convolutional, and attention-based networks improve sequence modelling, enabling longer forecasting horizons and better handling of irregular fluctuations [21,22]. However, a recent large-scale comparison of statistical, machine-learning, neural-network, and graph-based models shows that increasing model complexity does not guarantee superior highway traffic-flow forecasts across all datasets and scenarios [23]. Recent studies further incorporate task-specific traffic mechanisms into attention-based forecasting. Qian et al. formulate short-term highway congestion prediction as a multi-task problem that jointly models traffic flow and event-induced capacity variability using an iTransformer-based architecture [24]. Comprehensive surveys document this rapid shift from shallow predictors toward deep architectures and the growing role of learned representations [3,20]. In parallel, sensing-oriented studies examine the data layer on which these models operate, addressing detector performance, noisy observations, and quality control so that downstream predictors receive reliable inputs [10,25]. These purely temporal models, however, treat each sensor independently and ignore the spatial propagation of congestion along the road network, which is the principal source of predictability over short horizons [3]. This limitation motivates the integration of temporal modules with graph-based spatial operators, an approach that now characterizes the field [26].

### 2.2. Spatio-Temporal Graph Learning for Traffic Sensor Networks

Spatio-temporal graph neural networks bridge this gap by combining temporal modelling with graph convolution. Diffusion convolutional recurrent networks model traffic as a diffusion process on a directed sensor graph [4], spatio-temporal graph convolutional networks combine graph and temporal convolution for efficient prediction [5], and dilated-convolution variants extend the receptive field to capture long-range dependencies [6]. A central design choice in these models is how the sensor graph is constructed [27]. Early graph-based models use a fixed adjacency derived from road-network distance, which is interpretable but cannot reflect functional correlations that deviate from physical proximity [5]. Beyond road traffic-state forecasting, spatial relationships constructed from geographic and activity similarity have also been incorporated into spatio-temporal graph convolutional models for station-level bicycle-ridership prediction [28]. This application indicates that graph construction can benefit from functional similarity rather than physical distance alone.

Adaptive-graph methods instead learn the dependency structure end-to-end through trainable node embeddings, allowing data-driven discovery of latent relations without a predefined matrix [7,8]; multivariate graph-learning models extend this idea by jointly inferring the graph and the forecaster from the data alone [29]. Related research has explicitly estimated propagation strength between urban intersections and mined influential interaction patterns from the resulting traffic graph [30]. Recent forecasting architectures have also combined graph-based spatial modelling with Transformer-based temporal modelling for multi-step passenger-flow prediction across interconnected stations [31]. Other developments incorporate traffic knowledge identified by large language models into spatio-temporal graph forecasters [32], while attention-based graph convolution has been extended to network-level crash-risk evaluation using multiple traffic-related information sources [33]. The latter applications address different prediction targets but further demonstrate the ability of graph-based models to represent complex network-wide dependencies. Recent traffic-state forecasting models additionally employ multiview adaptive Transformer–GRU modelling, multi-scale hierarchical and local attention, and auxiliary-information-aware sparse graph convolution [34,35,36]. These methods, however, generally assume that the sensor set is available during model training.

A common thread across adaptive and dynamic approaches is that each sensor is associated with a globally learned identity embedding optimized over the full training network. This design is highly effective for sensors observed during training but offers no principled way to represent a sensor absent from the training set: a node absent during training has no corresponding sensor-identity embedding, and its row in the learned adjacency is undefined [7,12]. Spatio-temporal kriging and inductive variants partially mitigate this by aggregating signals from neighbouring observed sensors to estimate values at unmonitored points [11]. Prediction under sparse spatial observations has also been studied in transportation hubs, where pedestrian-dynamics priors and hypergraph attention are combined to estimate network-wide passenger distributions from limited monitoring data [37]. This setting is related to the present study through its limited-observation constraint, but it predicts spatial passenger distributions rather than adapting a pre-trained traffic forecaster to sensor locations unseen during training. In practice, however, these methods still require a substantial pool of well-observed neighbours, and they treat the dependency structure as a by-product of node features rather than as a quantity to be explicitly inferred from a small target sample. When only a few days of support data are available for the new sensors themselves, the reconstructed graph is unreliable, and the very spatial structure on which graph convolution depends becomes the weakest link.

### 2.3. Transfer Learning and Domain Adaptation for Traffic Forecasting

Transfer learning and domain adaptation reduce the dependence of traffic forecasting models on abundant target data. Recent distribution-shift-aware approaches also improve the use of long-term historical data by accounting for differences between historical and current traffic distributions [38]. This direction is complementary to the present focus on adapting representations and spatial dependencies for sensor nodes absent during training. Cross-city pipelines pre-train on a data-rich source network and transfer the learned representations to a sparse target, matching regions by demand similarity to reduce negative transfer [13]. Recent reviews of cross-city transfer in smart cities and sustainable transportation further emphasize that transferability depends on data availability, urban heterogeneity, and task alignment [15]. Domain-adaptation variants further align the feature distributions of source and target through adversarial or statistical objectives, improving robustness when the two networks differ [14]. These methods can be effective when source and target are reasonably similar, but their performance degrades under pronounced distribution shift, and they typically assume a target dataset large enough for stable fine-tuning. Most cross-city designs also operate at the level of an entire network or grid, transferring a global representation rather than reasoning about individual sensors; the granularity at which transfer is performed therefore does not match the granularity at which new detectors are physically deployed. Crucially, such schemes offer no guarantee of *fast* adaptation from only a few days of data: the transfer is performed once, in bulk, rather than as a rapid per-sensor update. This leaves open the operationally important regime in which a handful of new sensors must become productive within hours of installation, with neither a similar source city nor a large target history to draw on.

### 2.4. Meta-Learning and Few-Shot Traffic Prediction

Meta-learning offers a more direct route to data efficiency. Optimization-based methods such as model-agnostic meta-learning and its first-order variants learn an initialization from which new tasks can be solved in a few gradient steps [39,40]. Traffic-specific methods apply this idea to cross-city knowledge transfer and few-sample station prediction and demonstrate clear gains over conventional fine-tuning [12,14,41,42]. Nevertheless, existing meta-learning approaches for traffic typically meta-learn only the forecasting parameters while keeping the graph structure either fixed or globally shared across tasks; the sensor dependency graph is not conditioned on the target support set, so the spatial structure exploited during adaptation is not tailored to the actual sensors being deployed [12,42]. Where a graph module is attached to a meta-learner, it is generated from global parameters and merely fine-tuned, rather than synthesized from the descriptors of the specific support set at hand [14]. Consequently, the two distinct sources of cold-start difficulty—unfamiliar temporal dynamics and an unknown spatial dependency structure—are addressed only partially. Closing this gap, by jointly adapting the forecasting parameters and a support-conditioned, sensor-level dependency structure within a single fast-adaptation loop, is precisely the objective of this paper. Table 1 summarizes representative method groups along the dimensions most relevant to cold-start sensor deployment.

## 3. Problem Definition

This section formalizes cold-start traffic state forecasting at sensor locations unseen during model training. The notation is summarized in Table 2, and the split rules below are stated so that they can be reproduced directly in code.

### 3.1. Traffic Sensor Network and Forecasting Task

We model a traffic sensor network as a graph G=(V,E,A), where V is the set of N=|V| sensors, E is the set of edges, and $A\in R^{N\times N}$ is an adjacency matrix encoding directed inter-sensor dependencies. At time step *t*, the network observation is $X_{t}\in R^{N\times C}$, where *C* is the number of measured channels (e.g., speed, flow, occupancy). Data are sampled every 5 min.

Given a historical window of length *P*, multi-step traffic forecasting learns a mapping from the past *P* observations to the next *Q* observations,

(1) $$f_{\Theta }:\;(X_{t-P+1},\ldots ,X_{t})\;⟼\;({\hat{X}}_{t+1},\ldots ,{\hat{X}}_{t+Q}),$$

with parameters Θ. We use P=Q=12, i.e., one hour of history to predict the next hour; with 5-min sampling, the  third, sixth, and twelfth predicted steps correspond to the 15, 30, and 60 min horizons reported in the experiments. For brevity, we write the input window as $X_{t}\in R^{P\times N\times C}$ and the prediction target as $Y_{t}\in R^{Q\times N\times C}$.

### 3.2. Source Sensors, Target Sensors, and the Cold-Start Setting

The core difficulty addressed in this work is that not all sensors are data-rich. We therefore partition the sensor set into a *source* subset and a *target* subset,

(2) $$V=V^{\mathrm{src}}\cup V^{\mathrm{tgt}},\;V^{\mathrm{src}}\cap V^{\mathrm{tgt}}=⌀.$$

Source sensors $V^{\mathrm{src}}$ have a long observation history and are used for meta-training. Target sensors $V^{\mathrm{tgt}}$ represent locations excluded from model training. Only *K* days of observations are available for these target nodes, where K∈{1,3,7}. This *K*-day period forms the support data used for adaptation.

In the intra-network new-sensor evaluation used for benchmarking, all sensors are physically present in the benchmark datasets. Target-node identities and observations are withheld during meta-training to reproduce the information constraint faced when a pre-trained model encounters previously unseen sensor locations. This protocol is a controlled proxy for incremental network expansion and does not provide direct field evidence from sensor locations added after model training. It reproduces the practical constraint that a sensor location absent during model training has only limited local observations available for adaptation. Because our graph learner (Section 4.2) generates sensor representations from support descriptors rather than from a fixed table of sensor-ID embeddings, the trained model transfers to a different target node set without architectural change; the benchmark protocol is therefore a controlled and reproducible proxy for the variable-*N* deployment case.

We consider two scenarios. In the held-out-node setting, $V^{\mathrm{tgt}}$ is a randomly selected subset of sensors excluded from training, representing incremental densification of an existing network. In the optional new-region setting, $V^{\mathrm{tgt}}$ is a spatially contiguous cluster (obtained by geographic or graph-based partitioning) disjoint from the source region, reflecting expansion into a new district. The new-region setting is treated as a supplementary, more challenging variant rather than the central claim.

### 3.3. Few-Shot Tasks and the No-Leakage Protocol

Following the episodic meta-learning convention, adaptation is organized into tasks. A task $T_{i}=\left(S_{i},Q_{i}\right)$ is associated with a group of target sensors and consists of a support set $S_{i}$ and a query set $Q_{i}$. The support set contains (X,Y) windows drawn from the *K*-day support period and is used for inner-loop adaptation; the query set contains windows drawn from a later, held-out period and is used to evaluate the adapted model and to drive the outer-loop meta-update. During meta-training, pseudo-target sensors are repeatedly sampled from $V^{\mathrm{src}}$ to form episodic tasks, and their *K*-day support windows and subsequent query windows reproduce the cold-start situation that the held-out real target sensors will pose at test time. This induces a task distribution p(T).

The meta-objective is to learn an initialization Θ that, after a few gradient steps on $S_{i}$, performs well on $Q_{i}$:

(3) $$\min_{\Theta }\;E_{T_{i}∼p\left(T\right)}\left[\;L_{Q_{i}}\;\left(U_{S_{i}}\left(\Theta \right)\right)\right],$$

where $U_{S_{i}}$ denotes the inner-loop adaptation operator defined in Section 4.

At adaptation and test time, the model uses observations from all available source and target nodes as a network-level input. It jointly produces future traffic-state predictions across the graph, while the adaptation loss and reported evaluation metrics are computed on the held-out target nodes. Source nodes therefore provide spatial context; they are not separate predictors for the target nodes.

To guarantee a realistic and reproducible evaluation, we enforce three rules. (i) Temporal causality: for every task, the support window strictly precedes the query window in time, so adaptation never uses future information. (ii) Query isolation: query targets $Y\in Q_{i}$ are used only for evaluation and the outer-loop meta-update, never for inner-loop adaptation. (iii) Normalization hygiene: the global standardization statistics (mean and standard deviation) used to scale model inputs and outputs are fitted on the source/training data only and applied unchanged elsewhere; statistics derived from the target support set enter the model exclusively through the support-set encoder as features, and never redefine the global normalization. All main results are reported as mean ± standard deviation over multiple random source/target splits.

## 4. Proposed Methodology

**Figure 1:**
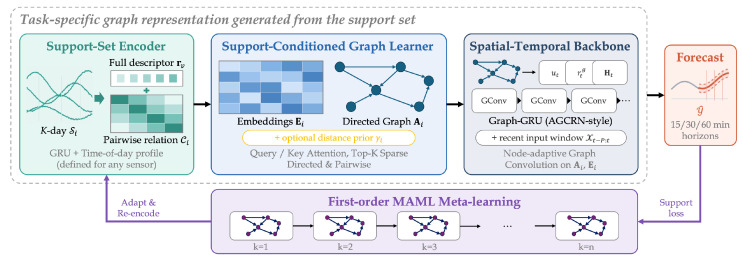
Overview of the proposed SC-SAMG framework. A *K*-day target support set is encoded into per-sensor descriptors and a pairwise relation matrix, from which the support-conditioned graph learner synthesizes task-specific embeddings $E_{i}$ and a directed adjacency $A_{i}$. The spatio-temporal backbone forecasts on this graph, and a first-order meta-learning loop adapts both the forecasting and graph-adaptation parameters from a few support steps, regenerating the graph at each step.

The proposed framework, support-conditioned sensor-adaptive meta-graph learning (SC-SAMG), turns a small target support set into a task-specific directed graph and a rapidly adapted forecaster. It has three components: (i) a support-set encoder that compresses limited target observations into per-sensor descriptors and a pairwise relation matrix; (ii) a support-conditioned graph learner that synthesizes task-specific sensor embeddings and a directed adjacency from those quantities; and (iii) a spatio-temporal backbone that forecasts on the synthesized graph. The three components are wrapped in a first-order meta-learning loop so that both the forecasting parameters and the graph-adaptation parameters are optimized for fast adaptation, with the graph regenerated at every adaptation step. Figure 1 shows the overall architecture. In plain terms, the support-set encoder first summarizes what each cold-start sensor has recently observed; the graph learner then converts these summaries into sensor representations and a dependency graph used by the forecaster. SC-SAMG is a single network-level forecasting model. It does not construct or train an independent predictor for each sensor.

### 4.1. Support-Set Encoder

For each target sensor *v*, the encoder extracts a compact descriptor from its *K*-day support observations. We combine hand-crafted statistical descriptors, which are stable even for very small *K*, with a learned temporal representation. The statistical descriptor is

(4) $$s_{v}=\left[\mu_{v},\;\sigma_{v},\;\tau_{v},\;p_{v},\;\rho_{v}\right],$$

where $\mu_{v}$ and $\sigma_{v}$ are the mean and standard deviation of the observed series, $\tau_{v}$ is a robust (Theil–Sen) trend slope normalized by the window length, $p_{v}\in R^{T_{d}}$ is a time-of-day profile obtained by averaging the series into $T_{d}=48$ half-hourly bins (a deliberately coarse resolution that remains estimable when K=1), and $\rho_{v}$ is the missing rate. In parallel, a lightweight shared temporal encoder (a gated recurrent unit) summarizes the raw support sequences into $z_{v}={\mathrm{TempEnc}}_{\phi_{e}}\left(\mathrm{support}\;\mathrm{windows}\;\mathrm{of}\;v\right)\in R^{d_{z}}$. The full descriptor is the concatenation $r_{v}=\left[s_{v};z_{v}\right]$, and stacking over sensors yields $R_{i}\in R^{N\times d_{r}}$.

Node-wise descriptors alone cannot express how two sensors interact, yet traffic dependency is inherently relational. We therefore also compute a task-level pairwise relation matrix $C_{i}\in R^{N\times N}$ directly from the support set, whose entry $C_{i,uv}$ measures the support-period association between sensors *u* and *v*.

In the main experiments, $C_{i}$ is constructed from the target support set using lagged Pearson correlation. For each sensor pair (u,v), the support series are standardized using observed values within the support window, and correlations are computed only over jointly observed timestamps. We evaluate lags ℓ∈{0,1,2,3}, corresponding to 0–15 min under 5 min sampling, and define

(5) $$C_{i,uv}=\max_{\ell \in \{0,1,2,3\}}\max\left\{0,\mathrm{corr}\left(x_{u}\left(t+\ell \right),x_{v}\left(t\right)\right)\right\}.$$

Undefined correlations caused by missing or constant series are set to zero, negative correlations are clipped to zero, and diagonal entries are set to zero because self-influence is supplied by the identity term in the graph convolution. Because both $r_{v}$ and $C_{i}$ are derived purely from the target support data, the encoder applies identically to sensors never seen during training.

### 4.2. Support-Conditioned Graph Learner

Instead of looking up a globally optimized identity embedding for each sensor—which is undefined for unseen nodes—we generate task-specific sensor embeddings from the support descriptors:

(6) $$e_{v}={\mathrm{MLP}}_{\phi_{g}}\left(r_{v}\right)\in R^{d},\;E_{i}=\left[e_{1};\ldots ;e_{N}\right]\in R^{N\times d}.$$

To model the directed nature of traffic propagation, the affinity is asymmetric: separate query and key projections of the embeddings are combined with the pairwise support relations,

(7) $$\begin{array}{cc} Q_{i}=E_{i}W_{q},\;K_{i} & =E_{i}W_{k}, \end{array}$$

(8) $$\begin{array}{cc} M_{i} & =\mathrm{ReLU}\;\left(\frac{Q_{i}K_{i}^{⊤}}{\sqrt{d}}+\eta \;C_{i}\right), \end{array}$$

where $W_{q},W_{k}\in R^{d\times d}$ and η weights the data-driven relations. The diagonal of $M_{i}$ is masked to −∞ so that self-influence is supplied separately by the identity term in the backbone; optionally, only the top-*k* entries of each row are retained:

(9) $$M_{i,uv}\leftarrow -\infty \;\mathrm{if}\;v\notin N_{k}\left(u\right),$$

which sparsifies the graph and lowers complexity. The directed adjacency is obtained by row-wise softmax and blended with an optional distance prior through a task-adaptive gate $\gamma_{i}$:

(10) $$\begin{array}{cc} A_{i} & =\gamma_{i}\;{\mathrm{softmax}}_{\mathrm{row}}\left(M_{i}\right)+\left(1-\gamma_{i}\right)\;{\hat{A}}_{\mathrm{dist}}, \end{array}$$

(11) $$\begin{array}{cc} \gamma_{i} & =\sigma \;\left(w_{\gamma }^{⊤}{\bar{r}}_{i}+b_{\gamma }\right),\;{\bar{r}}_{i}=\frac{1}{N}\sum_{v}r_{v}. \end{array}$$

Equations (6)–(11) realize the mapping $A_{i}=g_{\phi_{g}}\left(S_{i},{\hat{A}}_{\mathrm{dist}}\right)$: the adjacency is a function of the support set, so different deployments produce different, sensor-appropriate and direction-aware dependency structures. When no reliable distance prior is available (e.g., a brand-new corridor), the gate can drive $\gamma_{i}\;\to \;1$ and the graph is inferred from support data alone. Both $E_{i}$ and $A_{i}$ are regenerated and refined by the inner-loop adaptation of Section 4.4, so the graph is not generated once but is itself meta-adapted.

### 4.3. Spatio-Temporal Backbone

The forecaster is an AGCRN-style recurrent graph encoder [7]. We emphasize that the backbone is only the forecasting engine; the contribution of this paper is that its graph and node parameters are support-conditioned and meta-adapted, rather than tied to a single set of globally learned embeddings. Concretely, the node-adaptive graph convolution uses the synthesized directed graph $A_{i}$ and embeddings $E_{i}$:

(12) $$\mathrm{GConv}\left(H\right)=\left(I_{N}+A_{i}\right)\;H\;\left(E_{i}W\right)+E_{i}b,$$

where $W\in R^{d\times C_{\mathrm{in}}\times C_{\mathrm{out}}}$ and $b\in R^{d\times C_{\mathrm{out}}}$ are shared weight/bias pools, so each sensor obtains its own convolution parameters through its embedding $e_{v}$ without storing *N* separate weight matrices, and the identity term $I_{N}$ supplies the self-loop. The convolution is embedded in a gated recurrent cell, with [·,·] denoting channel concatenation and ⊙ the Hadamard product.

(13) $$\begin{array}{cc} u_{t} & =\sigma \;\left({\mathrm{GConv}}_{u}\left(\left[X_{t},H_{t-1}\right]\right)\right), \end{array}$$

(14) $$\begin{array}{cc} r_{t}^{g} & =\sigma \;\left({\mathrm{GConv}}_{r}\left(\left[X_{t},H_{t-1}\right]\right)\right), \end{array}$$

(15) $$\begin{array}{cc} {\tilde{H}}_{t} & =\tanh\;\left({\mathrm{GConv}}_{h}\left(\left[X_{t},r_{t}^{g}⊙H_{t-1}\right]\right)\right), \end{array}$$

(16) $$\begin{array}{cc} H_{t} & =u_{t}⊙H_{t-1}+\left(1-u_{t}\right)⊙{\tilde{H}}_{t}. \end{array}$$

The final hidden state is projected by a linear head to produce all *Q* horizons simultaneously. $\hat{Y}=W_{o}H_{P}+b_{o}$.

### 4.4. Meta-Learning and Optimization

Let $\Theta =\{\phi_{e},\phi_{g},\theta \}$ collect the encoder, graph-learner, and backbone parameters. We adapt Θ with first-order MAML (FO-MAML) [39,40], which avoids second-order derivatives and is therefore well suited to deployment on modest hardware. For task $T_{i}$, the inner loop performs *n* gradient steps on the support loss, and the embeddings and adjacency are recomputed from the current adapted parameters at each step:

(17) $$\begin{array}{cc} E_{i}^{\left(k\right)},A_{i}^{\left(k\right)} & =g_{\phi_{g}^{\left(k\right)}}\;\left({\mathrm{Encoder}}_{\phi_{e}^{\left(k\right)}}\left(S_{i}\right),{\hat{A}}_{\mathrm{dist}}\right), \end{array}$$

(18) $$\begin{array}{cc} \Theta_{i}^{\left(k+1\right)} & =\Theta_{i}^{\left(k\right)}-\alpha \;\nabla_{\Theta_{i}^{\left(k\right)}}\;L_{S_{i}}\;\left(\Theta_{i}^{\left(k\right)}\right),\;\Theta_{i}^{\left(0\right)}=\Theta , \end{array}$$

for k=0,…,n−1, where the support loss in step *k* is evaluated with $A_{i}^{\left(k\right)}$. The gradient therefore flows through both the forecasting parameters θ and the graph-adaptation parameters $\left\{\phi_{e},\phi_{g}\right\}$, so the synthesized graph is genuinely refined for the task. The outer loop updates the shared initialization using the query loss at the adapted parameters; under the first-order approximation, the dependence of $\Theta_{i}^{\left(n\right)}$ on the inner trajectory is dropped.

(19) $$\nabla_{\Theta }\;L_{Q_{i}}\;\left(\Theta_{i}^{\left(n\right)}\right)\;\approx \;\nabla_{\Theta_{i}^{\left(n\right)}}\;L_{Q_{i}}\;\left(\Theta_{i}^{\left(n\right)}\right),$$

yielding the meta-update over a meta-batch B of tasks.

(20) $$\Theta \;\leftarrow \;\Theta -\beta \sum_{T_{i}\in B}\nabla_{\Theta_{i}^{\left(n\right)}}\;L_{Q_{i}}\;\left(\Theta_{i}^{\left(n\right)}\right),$$

where the query loss is itself evaluated with the adapted graph $A_{i}^{\left(n\right)}$ produced by Equation (17) at k=n.

The primary loss is the masked mean absolute error over the target sensors, which ignores missing or invalid entries—essential when target sensors have non-trivial missing rates:

(21) $$L_{\mathrm{MAE}}=\frac{1}{\left|\Omega \right|}\sum_{(q,v,c)\in \Omega }\left|{\hat{Y}}_{q,v,c}-Y_{q,v,c}\right|,$$

where Ω indexes observed entries on target sensors [4,6]. To encourage a parsimonious and physically plausible dependency structure, we add optional regularizers on the synthesized graph. Because $A_{i}$ is row-normalized, an $\ell_{1}$ penalty would be (almost) constant and ineffective; we instead use a row-entropy penalty that concentrates each sensor’s attention on a few neighbours, together with a Dirichlet smoothness term:

(22) $$L=L_{\mathrm{MAE}}+\lambda_{1}\;\sum_{u}H\;\left(A_{i,u:}\right)+\lambda_{2}\;\;\sum_{u,v}A_{i,uv}\;{∥e_{u}-e_{v}∥}_{2}^{2},$$

with $H\left(A_{i,u:}\right)=-\sum_{v}A_{i,uv}\log\left(A_{i,uv}+\epsilon \right)$; the third term discourages strong edges between dissimilar sensors. Both regularizers are disabled by default ($\lambda_{1}=\lambda_{2}=0$) and studied in the ablation; when sparsity is desired, the top-*k* truncation of Equation (9) offers a hard alternative to the entropy term. Algorithms 1 and 2 give the meta-training and deployment procedures.
**Algorithm 1** SC-SAMG meta-training**Require:** Source data $D^{\mathrm{src}}$; learning rates α,β; inner steps *n*
  1:Initialise $\Theta =\{\phi_{e},\phi_{g},\theta \}$  2:**while** not converged **do**  3:      Sample a meta-batch B of tasks $T_{i}=\left(S_{i},Q_{i}\right)$ with pseudo-target sensors from $V^{\mathrm{src}}$  4:      Precompute pairwise relations $C_{i}$ from $S_{i}$     ▹ data-only, fixed across inner steps  5:      **for** each task $T_{i}\in B$ **do**  6:            $\Theta_{i}^{\left(0\right)}\;\leftarrow \;\Theta$  7:            **for** k=0 to n−1 **do**  8:                  $R_{i}^{\left(k\right)}\;\leftarrow \;{\mathrm{Encoder}}_{\phi_{e}^{\left(k\right)}}\left(S_{i}\right)$;   $E_{i}^{\left(k\right)},A_{i}^{\left(k\right)}\;\leftarrow \;g_{\phi_{g}^{\left(k\right)}}\left(R_{i}^{\left(k\right)},{\hat{A}}_{\mathrm{dist}},C_{i}\right)$      ▹ Equation (6)–(11)  9:                  $\Theta_{i}^{\left(k+1\right)}\;\leftarrow \;\Theta_{i}^{\left(k\right)}-\alpha \nabla_{\Theta_{i}^{\left(k\right)}}L_{S_{i}}\left(\Theta_{i}^{\left(k\right)}\right)$                             ▹ Equation (18), using $A_{i}^{\left(k\right)}$10:            **end for**11:            Regenerate $E_{i}^{\left(n\right)},A_{i}^{\left(n\right)}$ from $\Theta_{i}^{\left(n\right)}$ and evaluate $L_{Q_{i}}\left(\Theta_{i}^{\left(n\right)}\right)$12:      **end for**13:      $\Theta \;\leftarrow \;\Theta -\beta \sum_{T_{i}\in B}\nabla_{\Theta_{i}^{\left(n\right)}}L_{Q_{i}}\left(\Theta_{i}^{\left(n\right)}\right)$                                                  ▹ Equation (19)–(20)14:**end while**15:**return** meta-learned initialisation Θ


**Algorithm 2** SC-SAMG adaptation to unseen sensor locations or regions.**Require:** Meta-learned Θ; *K*-day target support set $S^{\mathrm{tgt}}$; inner steps *n*
1:Precompute pairwise relations C from $S^{\mathrm{tgt}}$2:$\Theta^{\prime }\;\leftarrow \;\Theta$3:**for** k=1 to *n* **do**4:      $R\;\leftarrow \;{\mathrm{Encoder}}_{\phi_{e}^{\prime }}\left(S^{\mathrm{tgt}}\right)$;   $E,A\;\leftarrow \;g_{\phi_{g}^{\prime }}\left(R,{\hat{A}}_{\mathrm{dist}},C\right)$                     ▹ regenerate with current $\phi^{\prime }$5:      $\Theta^{\prime }\;\leftarrow \;\Theta^{\prime }-\alpha \nabla_{\Theta^{\prime }}L_{S^{\mathrm{tgt}}}\left(\Theta^{\prime }\right)$6:**end for**7:Regenerate E,A from $\Theta^{\prime }$8:**return** adapted network-level model $f_{\Theta^{\prime }}$ for forecasting at the target nodes


### 4.5. Complexity and Distinction from AGCRN

Let *H* denote the hidden width. Forming the asymmetric affinity in Equation (8) costs $O(N^{2}d)$. With a dense graph, each graph-convolution step is $O(N^{2}H+Nd\;C_{\mathrm{in}}C_{\mathrm{out}})$, the first term coming from the adjacency–hidden product and the second from the node-adaptive weight generation; with the top-*k* sparsification of Equation (9), the adjacency–hidden product drops to O(kNH), giving $O(kNH+Nd\;C_{\mathrm{in}}C_{\mathrm{out}})$ per step. Meta-training over a meta-batch of *B* tasks with *n* inner steps and an *L*-step recurrence therefore scales as $O\;\left(B\;n\;L\;(kNH+Nd\;C_{\mathrm{in}}C_{\mathrm{out}})\right)$ in the sparse case—the same order as the underlying backbone up to the constant factor *n*. Deployment (Algorithm 2) requires only *n* inexpensive support steps on the target sensors, which is the property that makes the method attractive for edge or roadside-unit execution.

AGCRN learns a single global node-embedding matrix and one symmetric, data-adaptive graph shared across the whole training network; these are fixed after training and are undefined for sensors that did not appear during training. In SC-SAMG, (i) the node embeddings $E_{i}$ and the directed adjacency $A_{i}$ are functions of the target support set—node descriptors plus pairwise relations—rather than free global parameters, so they are defined for unseen sensors; (ii) they are produced by a dedicated support-set encoder and a query–key graph learner; and (iii) they are meta-adapted and regenerated at every inner step together with the forecaster through Equations (17)–(20). AGCRN is thus a special case of the backbone used inside SC-SAMG, not the contribution itself; the method is a support-conditioned, direction-aware graph-adaptation framework rather than a combination of AGCRN and MAML.

## 5. Experiments

This section evaluates SC-SAMG under deployment-oriented conditions: held-out-node and unseen-region adaptation, sensitivity to the support size, an ablation of each component, adaptation efficiency, and robustness to sensor outages. Unless otherwise stated, the main new-sensor results are reported as mean ± standard deviation over ten random source/target splits. Experiments with different protocols, such as efficiency and outage analyses, specify their split counts separately.

### 5.1. Experimental Setup

#### 5.1.1. Datasets and Settings

We evaluate four widely used loop-detector benchmarks spanning two traffic variables. For speed forecasting, we use METR-LA (207 sensors on Los Angeles highways, four months, March–June 2012) and PEMS-BAY (325 sensors in the San Francisco Bay Area, six months, January–May 2017). For flow (vehicle count) forecasting, we use PEMS04 (307 sensors, San Francisco Bay Area, two months, January–February 2018) and PEMS08 (170 sensors, San Bernardino, two months, July–August 2016), both released by [44]. All four are sampled every 5 min. We use an input length P=12 and a forecast length Q=12, and report the 15, 30, and 60 min horizons (the third, sixth, and twelfth predicted steps). Inputs and outputs are standardized with statistics fitted on the source training data only, following the no-leakage protocol of Section 3. Road-network distance information is treated as an optional physical prior rather than a required input to SC-SAMG. When a reliable sensor-to-sensor distance matrix is available from the corresponding benchmark release, we use it only to construct the optional distance prior ${\hat{A}}_{\mathrm{dist}}$ and, for the flow datasets, to define geographic region splits. When such a prior is removed or unavailable, the generated graph is inferred entirely from the support-conditioned sensor descriptors and pairwise support relations. To separate the contribution of this physical prior from the proposed support-conditioned graph learner, we report with- and without-distance-prior ablations in Section 5.5.

The held-out-node protocol provides a controlled test of whether a pre-trained network-level model can incorporate sensor locations excluded from training. The retained source nodes provide network context and the historical data required for meta-training. This protocol reproduces the information constraint of incremental network expansion without implying that the benchmark data were collected during an actual network expansion.

#### 5.1.2. Baselines and Metrics

We compare SC-SAMG against ten baselines chosen to cover parameter-free prediction, sequence fine-tuning, fixed-graph STGNNs, adaptive-graph STGNNs, inductive graph forecasting, traffic-specific meta-learning, and an internal no-graph control.

- Historical Average (HA): A parameter-free predictor returning each target sensor’s time-of-day mean over the support period.
- GRU-FT: A spatially agnostic gated recurrent network, pre-trained on the source sensors and fine-tuned on the *K*-day target support.
- DCRNN-FT [4]: A diffusion convolutional recurrent network, and a strong spatio-temporal graph baseline. To make it applicable to unseen sensors, we build its graph inductively from the support set (a row-normalized correlation graph defined for any node set) and share non-node-specific weights, then pre-train on the source network and fine-tune on the target support.
- STGCN-FT [5]: A classical fixed-graph spatio-temporal graph convolutional network. We construct the target-sensor graph from the available physical or support-derived relation matrix and fine-tune the non-node-specific forecasting parameters on the target support set.
- Graph WaveNet-FT [6]: A competitive adaptive-graph STGNN with dilated temporal convolution and learnable node-adaptive adjacency. For unseen target sensors, node embeddings are initialized from source-embedding statistics and then fine-tuned using only the target support set.
- MTGNN-FT [29]: A multivariate time-series graph neural network that jointly learns graph structure and temporal forecasting parameters. We adapt it to the new-sensor protocol by pre-training on source sensors and fine-tuning the graph learner and forecasting layers on the *K*-day target support.
- AGCRN-FT [7]: The adaptive-graph backbone with globally learned node embeddings, pre-trained on the source network and fine-tuned on the target support; embeddings of held-out target sensors are initialized from the mean source embedding before fine-tuning.
- IGNNK [11]: An inductive graph neural network for spatio-temporal kriging. It is included as an inductive graph baseline for unobserved or sparsely observed sensors; the graph used at test time is constructed from the same available support-derived relation information without using target query observations.
- ST-MetaNet [41]: A traffic-specific spatio-temporal meta-learning model that generates forecasting parameters from node and edge meta-knowledge. We train it under the same episodic support/query protocol as SC-SAMG and adapt it using only the target support set.
- FO-MAML (backbone): The proposed framework with its support-set encoder and first-order meta-learning loop, but with the learned graph replaced by an identity adjacency. It still serves unseen sensors through the support-conditioned embeddings, and therefore acts as the key control that isolates the contribution of the learned graph alone.

STGCN-FT, DCRNN-FT, Graph WaveNet-FT, MTGNN-FT, and AGCRN-FT test whether SC-SAMG remains competitive against fixed-graph and adaptive-graph STGNNs; IGNNK tests an inductive graph-reconstruction alternative; ST-MetaNet tests a traffic-specific meta-learning alternative; and FO-MAML isolates the contribution of the support-conditioned graph within our own framework. For fairness, all learnable baselines are trained on the source sensors and adapted using only the same *K*-day target support set. No target query observations are used for graph construction, model selection, or adaptation. Methods requiring target sensors to be present during training, fully observed target-node identity embeddings, or query-period target observations are not included because they violate the leakage-controlled new-sensor protocol.

Accuracy is measured by masked mean absolute error (MAE) and root mean squared error (RMSE), computed over the observed entries Ω on the target sensors:

(23) $$\begin{array}{cc} \mathrm{MAE} & =\frac{1}{\left|\Omega \right|}\;\;\sum_{(q,v,c)\in \Omega }\;\;\left|{\hat{Y}}_{q,v,c}-Y_{q,v,c}\right|, \end{array}$$

(24) $$\begin{array}{cc} \mathrm{RMSE} & ={\left(\frac{1}{\left|\Omega \right|}\;\;\sum_{(q,v,c)\in \Omega }\;\;{\left({\hat{Y}}_{q,v,c}-Y_{q,v,c}\right)}^{2}\right)}^{1/2}. \end{array}$$

We deliberately do not report MAPE. The cold-start evaluation retains genuine near-zero ground-truth values—heavily congested speeds of 1–2 mph and overnight flows of a few vehicles per interval—which, after masking out only the exactly-zero (missing) entries, leave percentage errors dominated by a handful of tiny denominators and render MAPE numerically ill-conditioned. MAE and RMSE, which are both well-behaved in this regime and standard in the traffic-forecasting literature [4,6], are used throughout.

#### 5.1.3. Implementation Details

All learnable methods share the same input/output dimensions and are trained using the Adam optimizer, with gradient clipping and early stopping on a source-only validation split, to ensure a fair comparison [45,46]. For SC-SAMG, we use a backbone hidden width of H=64, sensor-embedding dimension d=16, and a single-layer graph-GRU; the support encoder uses a GRU of width $d_{z}\;=\;32$ and a $T_{d}\;=\;48$-bin time-of-day profile. The graph learner uses query/key projections $W_{q},W_{k}\in R^{d\times d}$, top-*k* sparsification with k=20, a pairwise-relation weight η tuned on validation, and a LayerNorm on the support descriptor for training stability. Gradients are clipped to norm 5 in both the inner and outer loops, and inner-loop adaptation draws a mini-batch of at most 256 support windows (so that larger *K* does not increase adaptation cost). Meta-training uses n=3 inner steps by default (varied in Section 5.6.1), with Adam applied to both inner-loop adaptation and outer-loop meta-optimization. The corresponding learning rates are $\alpha \;=\;10^{-3}$ and $\beta \;=\;10^{-3}$, with a meta-batch of B=8 tasks. These values are selected on the source-only validation split from the ranges $\alpha \in \{10^{-4},5\times 10^{-4},10^{-3}\}$, $\beta \in \{10^{-4},10^{-3}\}$, and B∈{4,8,16}. The graph regularizers are disabled by default ($\lambda_{1}\;=\;\lambda_{2}\;=\;0$) and studied in the ablation. All hyperparameters, including the number of inner adaptation steps, are selected on the source validation tasks and never on the target query set, so target adaptation introduces no query leakage. Although Adam is used consistently in the present experiments, future work will compare alternative optimizers to assess their effects on convergence, training stability, and few-shot adaptation performance.

### 5.2. Main New-Sensor Results

**Figure 2:**
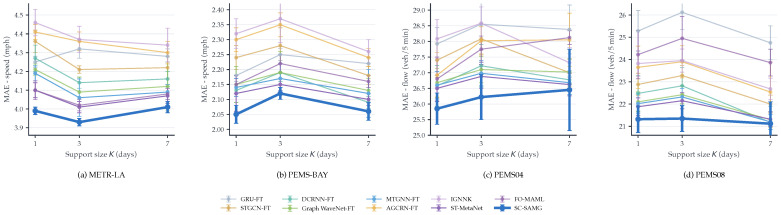
New-sensor MAE versus support size *K* on the four benchmarks (historical average omitted for scale). SC-SAMG (thick blue) lies below every baseline in all panels. Exact values and RMSE are in Table 3, Table 4, Table 5 and Table 6.

We use a 70/30 source/target split of the sensors: 70% are source sensors with full history for meta-training, and the remaining 30% are held-out target sensors that receive only K∈{1,3,7} days of support before being evaluated on a later query period. Splits are redrawn ten times and we report mean ± standard deviation. Table 3 and Table 4 report the horizon-averaged MAE and RMSE on the two speed networks, and Table 5 and Table 6 on the two flow networks; Figure 2 visualizes the same results as accuracy-vs-*K* curves. Statistical significance is assessed on the same ten source/target splits for all four datasets by comparing SC-SAMG with each baseline over 10splits×3K=30 matched split-support observations, using paired tests with Holm–Bonferroni correction (Section 5.3).

The support set for each target split is sampled as a contiguous calendar window preceding the query period, representing the sequential accumulation of observations before forecasting begins. Therefore, increasing *K* changes both the amount of available target data and the calendar composition of the support set. The resulting error is not expected to decrease monotonically with *K*. A one-day support window often reflects a coherent traffic regime, whereas a three-day window can straddle weekday/weekend boundaries or short-term demand transitions. A seven-day window is more likely to cover a complete weekly cycle and therefore better represents recurring traffic patterns. In addition, K=3 can be a transition point in fine-tuning: it provides enough data to move model parameters away from the source-trained initialization, but not enough coverage to fully represent the weekly distribution. This explains the localized K=3 spikes observed in several fine-tuning baselines, such as GRU-FT on METR-LA and DCRNN-FT on PEMS08. By contrast, the meta-learning methods are less affected, suggesting that the meta-learned initialization provides useful regularization under heterogeneous short support windows. A split-level inspection further confirms that the K=3 increases are concentrated in support windows crossing weekday/weekend boundaries or atypical demand transitions.

Table 3, Table 4, Table 5 and Table 6 show that SC-SAMG remains competitive after expanding the baseline set to include stronger fixed-graph, adaptive-graph, inductive, and meta-learning traffic forecasting models. Across all four benchmarks and all three support sizes, SC-SAMG achieves the lowest MAE and RMSE. This indicates that its advantage is not an artefact of comparing against weak fine-tuning baselines. Among the newly added methods, ST-MetaNet is generally the strongest competitor, followed by MTGNN-FT and Graph WaveNet-FT. At K=1, SC-SAMG reduces MAE over ST-MetaNet by 2.7% on METR-LA, 3.3% on PEMS-BAY, 2.5% on PEMS04, and 2.6% on PEMS08. At K=3, the corresponding reductions are 2.0%, 1.4%, 2.5%, and 3.6%. The margins become smaller at K=7, as expected, because additional target support data reduce the severity of the cold-start condition.

The speed benchmarks show that SC-SAMG remains strongest even when compared with recent adaptive-graph and meta-learning baselines. On METR-LA, SC-SAMG achieves the best MAE at all support sizes (3.99, 3.93, and 4.01) and also obtains the lowest RMSE (7.70, 7.68, 7.73), although ST-MetaNet and MTGNN-FT are close behind, confirming that the newly added baselines are indeed competitive. On PEMS-BAY, SC-SAMG is stronger in both MAE and RMSE across all support sizes, improving over ST-MetaNet from 2.12 to 2.05 at K=1, from 2.15 to 2.12 at K=3, and from 2.10 to 2.06 at K=7.

**Figure 3:**
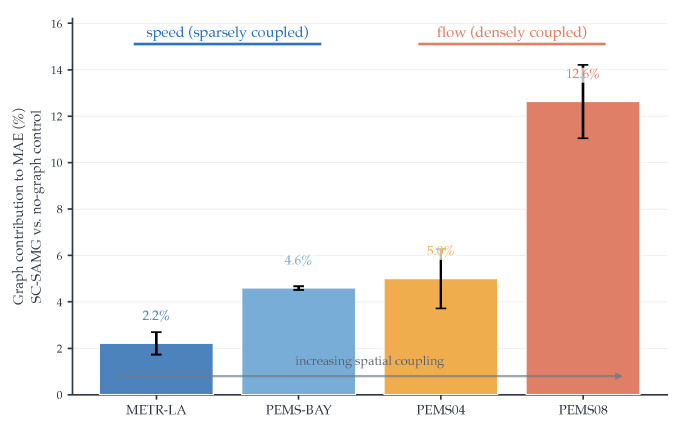
The learned graph is a density-dependent enhancement. Bars show the MAE improvement of SC-SAMG over the no-graph control (FO-MAML), averaged over K∈{1,3,7} (error bars: s.d. across *K*). The contribution is marginal on the sparsely coupled speed networks (≈2–4%) but large on the densely coupled flow network PEMS08 (≈13%).

The flow benchmarks further show where the support-conditioned graph is most useful. On PEMS04, SC-SAMG improves over the strongest newly added baseline, ST-MetaNet, by 2.5%, 2.5%, and 0.6% MAE at K=1,3,7, respectively. On PEMS08, SC-SAMG improves over ST-MetaNet by 2.6% and 3.6% at K=1 and K=3, respectively; at K=7, it remains slightly better than the strongest newly added baseline, MTGNN-FT (21.12 vs. 21.20, a 0.4% reduction). This agrees with the density-dependent pattern observed in Figure 3: when the network is denser and spatial propagation carries stronger predictive signal, generating the dependency graph from the target support set becomes more beneficial.

The expanded comparison also clarifies the limitations of different baseline families. STGCN-FT is constrained by its fixed graph. Graph WaveNet-FT and MTGNN-FT benefit from adaptive graph learning, but their learned node embeddings and graph parameters are still source-trained and are not explicitly generated for unseen target sensors. IGNNK can operate inductively, but its reconstruction-oriented objective is less effective for multi-step forecasting than end-to-end prediction models. ST-MetaNet is the closest competitor in most settings, especially on the sparse METR-LA network, but it does not explicitly synthesize a target-specific dependency graph from the support set. Overall, the results support the central claim that SC-SAMG improves cold-start traffic state forecasting at unseen sensor locations by jointly adapting sensor representations and the task-specific graph structure under limited target support.

### 5.3. Statistical Significance

To provide a unified assessment of statistical reliability, we repeat the new-sensor experiments over ten random source/target splits on all four datasets. For each dataset, support size, and baseline, the same split is used for SC-SAMG and the corresponding comparator, enabling paired testing. We aggregate the 10 splits × 3 support sizes into 30 matched split-support observations per comparison. Because the same support sizes and source/target splits are used for every method, the paired tests compare method differences under matched cold-start conditions rather than treating support sizes as unrelated datasets. We conduct both paired *t*-tests and Wilcoxon signed-rank tests. Holm–Bonferroni correction is applied across the ten baselines within each dataset to control the family-wise error rate. We additionally report the absolute Cohen’s $d_{z}$, computed as the absolute mean paired difference divided by the standard deviation of the paired differences. Table 7 shows that SC-SAMG significantly outperforms almost all baselines on all four datasets after correction. The only borderline case is the paired *t*-test against ST-MetaNet on PEMS04 ($p_{\mathrm{corr}}=0.058$), while the Wilcoxon test for the same comparison remains significant ($p_{\mathrm{corr}}=0.048$). This unified protocol replaces the previous dataset-specific significance check and provides a consistent statistical assessment across all benchmarks.

The statistical tests confirm the stability of the main conclusions. SC-SAMG significantly outperforms almost all baselines on all four datasets after Holm–Bonferroni correction under both paired *t*-tests and Wilcoxon signed-rank tests. The only borderline case is the paired *t*-test against ST-MetaNet on PEMS04 ($p_{\mathrm{corr}}=0.058$), although the corresponding Wilcoxon test remains significant ($p_{\mathrm{corr}}=0.048$). This indicates that the advantage over the strongest meta-learning baseline on PEMS04 is modest and affected by the larger split-to-split variability of the PEMS04 flow data, but the overall trend remains consistent. The effect sizes are also aligned with the empirical ranking in the main result tables: improvements over HA, IGNNK, and conventional fine-tuning baselines are large, while improvements over ST-MetaNet, MTGNN-FT, Graph WaveNet-FT, and FO-MAML are smaller but generally significant. These results provide a unified and more robust validation of SC-SAMG across all four benchmarks.

### 5.4. New-Region Adaptation

**Figure 4:**
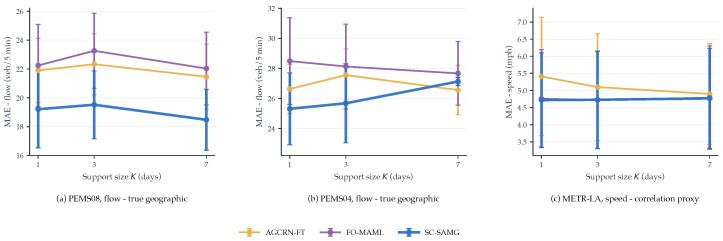
New-region adaptation: MAE versus *K* with an entire area held out (geographic clusters for the flow networks; a correlation proxy for METR-LA). SC-SAMG (blue) leads on the dense flow networks and ties within the large spread on METR-LA. Exact values in Table 8.

To assess transferability to an entire unseen area, we replace the random sensor split with a region split: an entire spatial cluster of sensors is held out as the target and receives only *K* days of support. This is a strictly harder setting that reflects expansion into a new district rather than densification of a known corridor. We use the two flow benchmarks as the primary test because they ship a road-network distance file: we apply spectral clustering to the distance affinity to obtain four true geographic regions and hold each out in turn. For METR-LA, which provides no coordinates, we fall back to clustering on training-period time-series correlation (a functional-region proxy) and report it as a supplementary check. Table 8 and Figure 4 report horizon-averaged MAE on all three networks.

To identify the data boundary of meta-adaptation, we further extend the METR-LA regional split to larger support sizes K∈{14,21,28}, in addition to the original K∈{1,3,7}. Table 9 compares AGCRN-FT, FO-MAML, and SC-SAMG under this extended support range.

The extended-*K* analysis identifies the data boundary of meta-adaptation. SC-SAMG is most beneficial in the strict few-shot regime: at K=1, it improves over AGCRN-FT by approximately 12.8% MAE on the METR-LA regional split. This advantage steadily decreases as more target-region data become available. AGCRN-FT reaches the one-day SC-SAMG performance after roughly two weeks of target support data and becomes statistically comparable to SC-SAMG after approximately three weeks. By K=28, AGCRN-FT slightly surpasses SC-SAMG, but the difference is only 0.05 MAE and remains well within the across-region standard deviation. This indicates that the meta-learned initialization does not cause meaningful negative transfer when abundant target data are available; rather, it becomes less necessary as conventional fine-tuning accumulates enough data to estimate target-region embeddings and graph parameters reliably. SC-SAMG therefore functions primarily as a cold-start accelerator that front-loads adaptation through meta-learning, rather than as a method expected to dominate indefinitely when abundant target-domain data are available.

New-region adaptation is harder than new-sensor adaptation, and the across-region variance is correspondingly larger. On the two flow networks, where the held-out clusters are genuine geographic regions, SC-SAMG is clearly the best method: on PEMS08, it lowers MAE by 12–16% relative to both AGCRN-FT and the no-graph control across all support sizes (e.g., 19.21 vs. 21.90/22.24 at K=1), and on PEMS04, it leads at K=1,3 (with AGCRN-FT marginally ahead only at K=7). Notably—and unlike the speed case—the learned graph contributes strongly here: SC-SAMG beats the FO-MAML control by 14–16% on PEMS08, mirroring the dense-network behaviour seen in the main flow results. This indicates that when an entire dense region must be served from a few days of data, inferring its dependency structure from the support set is especially valuable. On the supplementary METR-LA correlation-region split, the picture matches the sparse-speed regime of the main experiment: SC-SAMG and the FO-MAML control are statistically tied (e.g., 4.72 vs. 4.76 at K=1) and both clearly outperform AGCRN-FT (by 13% at K=1), with the graph’s marginal benefit again small. The large METR-LA spread (±1.4–1.7 in the few-shot region split) reflects strongly varying per-region difficulty. Within the original K≤7 few-shot range, increasing *K* barely changes the error, indicating that the residual is dominated by cross-region distribution shift rather than target-data scarcity. However, the extended-*K* analysis in Table 9 shows that when multiple weeks of target support become available, standard structural fine-tuning gradually closes the gap. Overall, new-region results reinforce the main finding: support-conditioned meta-adaptation transfers to entirely new areas, and the learned graph adds the most on dense networks.

### 5.5. Ablation Study

**Figure 5:**
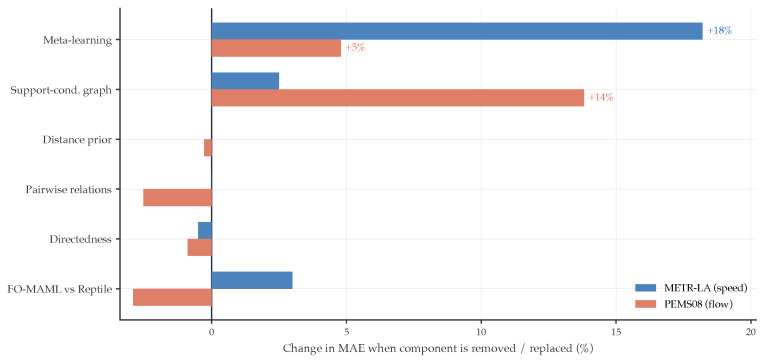
Ablation: percentage change in MAE when each component is removed or replaced (K=1). Meta-learning dominates on the sparse speed network and the support-conditioned graph on the dense flow network, while the distance prior and finer refinements stay within noise. Absolute values in Table 10.

We remove or replace one component at a time at K=1 (the hardest, cold-start regime), reporting horizon-averaged MAE and RMSE over ten splits on a sparse speed network (METR-LA) and a dense flow network (PEMS08) (Table 10). The variants are: (a) w/o meta-learning—the support-conditioned graph and backbone are trained jointly and then fine-tuned, without the FO-MAML loop; (b) w/o support-conditioned graph—the support-conditioned embeddings are kept but the learned graph is replaced by an identity adjacency (no inter-sensor message passing); (c) w/o distance prior—the physical road-network distance prior ${\hat{A}}_{\mathrm{dist}}$ is removed, leaving the graph to be inferred from support-conditioned descriptors and pairwise relations alone; (d) w/o pairwise relations (η=0); (e) symmetric graph—the query/key projections are tied so the affinity is undirected; and (f) Reptile in place of the FO-MAML outer update. Table 10 reports the absolute errors and Figure 5 the relative component contributions.

The ablation cleanly separates the contributions, and the two networks together explain when each matters. Meta-learning is essential on both: removing it raises MAE by 18.3% on METR-LA (3.99→4.72) and by 4.8% on PEMS08 (21.32→22.34), and the first-order MAML outer update is no worse than Reptile. The support-conditioned graph is where the two regimes diverge, exactly as the main results predict: replacing it with an identity adjacency costs only 2.5% MAE on the sparse speed network but a large 13.8% on the dense flow network (21.32→24.26), confirming that the learned graph is a density-dependent enhancement rather than incidental machinery.

The distance prior is a minor ingredient: removing it changes MAE by only 0.5% on METR-LA (3.99 vs. 4.01) and by −0.2% on PEMS08 (21.32 vs. 21.27), i.e., the data-driven graph already recovers most of the useful structure and the physical prior mainly aids interpretability and early-training stability. The finer graph refinements, pairwise relations, directedness, and the choice of outer update remain within one standard deviation of the full model on both datasets. On PEMS08, the pairwise-relation and Reptile variants are even marginally lower than the full model (20.78 and 20.70 vs. 21.32), which is within the experimental noise and suggests that these refinements are not consistently beneficial on the current loop-detector benchmarks. We therefore treat these components as optional rather than universally necessary. Overall, these results indicate that support-conditioned representation learning and fast meta-adaptation account for much of the cold-start performance observed here, whereas the learned graph provides a density-dependent refinement, with its clearest benefit on dense flow networks.

Because the pairwise support-relation matrix $C_{i}$ is estimated from only a small support set, we further examine whether the reported results depend on its specific construction. We conduct this sensitivity analysis at K=1, the most challenging cold-start setting, where $C_{i}$ is estimated from the least target information. Only the construction of $C_{i}$ is changed, while all other model components, hyperparameters, and source/target splits are kept fixed. Table 11 compares four schemes: removing the pairwise relation term, zero-lag Pearson correlation, the default lagged Pearson correlation over 0–15 min, and masked covariance.

Table 11 shows that SC-SAMG is not highly sensitive to the specific construction of the pairwise support-relation matrix. On METR-LA, the default lagged Pearson scheme matches the best MAE and gives the lowest RMSE, while the no-pairwise and zero-lag variants remain very close. On PEMS08, the no-pairwise variant is marginally better in this run, but the differences among all relation schemes are within or close to one standard deviation. This pattern is consistent with the component ablation in Table 10: pairwise relations act as a lightweight relational prior rather than the dominant source of cold-start performance. We retain lagged Pearson correlation as the default because it is simple, reproducible, and physically interpretable for short-range traffic propagation, while acknowledging that the main conclusions do not depend critically on this choice.

To address whether the use of road-network distance information creates an inconsistent experimental setting across datasets, we further conduct a direct with/without-prior comparison on all four benchmarks. In the without-prior variant, the distance-based adjacency prior ${\hat{A}}_{\mathrm{dist}}$ is removed, while the support encoder, pairwise relation matrix, generated graph learner, forecasting backbone, and meta-learning procedure are kept unchanged. Therefore, the comparison isolates the contribution of the optional physical prior from the contribution of the proposed support-conditioned graph learning mechanism.

Table 12 shows that the distance prior has a minor and non-dominant effect on the overall performance gain. Removing ${\hat{A}}_{\mathrm{dist}}$ changes MAE from 3.99 to 4.01 on METR-LA, from 2.05 to 2.07 on PEMS-BAY, from 25.85 to 26.10 on PEMS04, and from 21.32 to 21.27 on PEMS08. These changes are small relative to the standard deviations and do not alter the ranking or the main conclusions. This indicates that SC-SAMG does not rely on the physical distance prior to achieve its cold-start advantage. Instead, the main contribution comes from support-conditioned sensor representation and data-driven graph generation. The distance prior is therefore best interpreted as a complementary physical cue that can improve interpretability and stabilize graph learning when reliable distance information is available.

### 5.6. Deployment Efficiency and Robustness

This subsection examines how quickly the model adapts to unseen target nodes and how well it tolerates missing sensor observations at test time.

#### 5.6.1. Adaptation Efficiency

**Figure 6:**
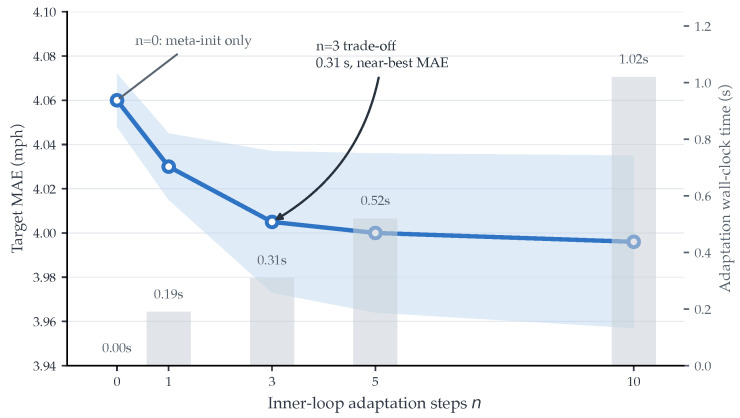
Adaptation efficiency on METR-LA (K=1): target MAE versus inner-loop adaptation steps *n* (mean ± std over ten splits). The meta-initialization (n=0) is already strong, and three steps recover essentially all of the attainable accuracy in under half a second.

On METR-LA at K=1 (the cold-start regime, three splits), we vary the number of inner-loop adaptation steps n∈{0,1,3,5,10} and record target MAE as a function of *n*, together with wall-clock adaptation time. The case n=0 corresponds to the meta-initialization applied without any target update. Figure 6 plots the resulting accuracy–step curve.

This experiment speaks directly to operational feasibility on the edge. The meta-initialization alone (n=0) already attains MAE 4.06, a single inner step lowers it to 4.03 in 0.19 s, and by n=3 the model reaches 4.005—within 0.2% of the best value at n=10 (3.996)—in only 0.31 s of wall-clock adaptation. In other words, three gradient steps taking under half a second recover essentially all of the attainable accuracy, whereas training an STGNN from scratch on the same data is orders of magnitude slower. A previously unseen sensor location can therefore be incorporated into the existing forecasting model using only a few inexpensive adaptation steps. The first-order design (Equations (19) and (20)) keeps each step inexpensive, reinforcing this argument.

#### 5.6.2. Missing-Sensor Robustness

To reduce over-reliance on a small number of generated hub sensors, we additionally test an outage-aware variant of SC-SAMG. During meta-training, we apply dropout to the generated adjacency matrix before row normalization:

(25) $${\tilde{A}}_{i}=\mathrm{RowNorm}\left(M⊙A_{i}\right),\;M_{uv}∼\mathrm{Bernoulli}\left(1-p_{e}\right),$$

where $p_{e}$ is the edge-dropout probability. This operation encourages the predictor to remain accurate when individual spatial dependencies are unavailable. We further add a soft column-degree penalty

(26) $$L_{\deg}=\lambda_{\deg}\sum_{v}{\left[\max\left(0,\sum_{u}A_{uv}-\tau \right)\right]}^{2},$$

where $\sum_{u}A_{uv}$ measures the total incoming dependency mass received by sensor *v*. This term penalizes excessive hub concentration and encourages the generated graph to distribute dependency across redundant neighbours. In the reported experiments, we set $p_{e}=0.2$, $\lambda_{\deg}=0.05$, and τ to 1.5 times the average column degree of the source-trained generated graph. These values were selected on held-out source validation episodes and then kept fixed across all outage ratios.

**Figure 7:**
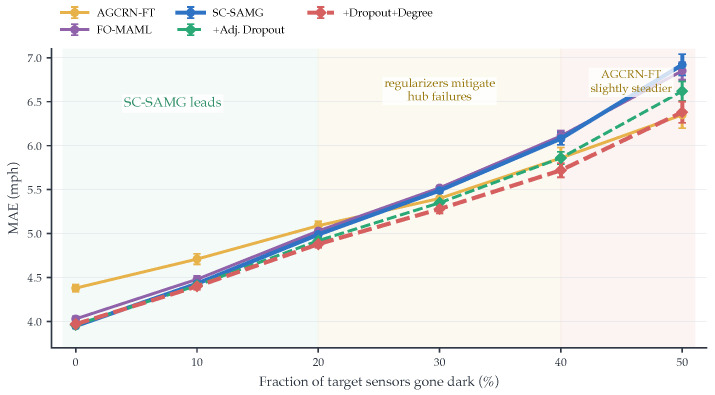
Sensor-outage robustness on METR-LA (K=3): MAE versus the fraction of target sensors that go dark. Original SC-SAMG leads under moderate outage, whereas adjacency dropout and degree regularization mitigate degradation under severe outages. Exact values are reported in Table 13 and Table 14.

Sensor faults and communication losses are routine in deployed networks, which is central to a sensing-oriented evaluation. We study a static sensor-outage setting on METR-LA (K=3): for each masking ratio in {10,20,30,40}% (plus a 0% reference), we randomly select that fraction of the target input sensors and set their test-time input to the standardized mean throughout the query period (“gone dark”); the support set used for adaptation is left intact, the support-conditioned graph is still built from it, and MAE is computed over all target sensors. We average over ten source/target splits and three outage masks per nonzero outage ratio. Table 13 and Figure 7 compare SC-SAMG against the AGCRN-FT and FO-MAML baselines.

We further evaluate whether the severe-outage degradation can be mitigated by the outage-aware regularizers. METR-LA is selected for this focused analysis because its relatively sparse topology makes hub concentration more pronounced and therefore provides a stringent test of the hub-dependency failure mode. Table 14 compares the original SC-SAMG with two regularized variants: adjacency dropout only and adjacency dropout with the soft column-degree penalty.

With no outage, SC-SAMG matches the corresponding main new-sensor result at K=3 and remains the most accurate method (3.93 vs. 4.02/4.36). It retains the lead up to a 20% outage (4.97 vs. 5.02/5.07). Beyond that point, the ranking reverses: the original SC-SAMG and the FO-MAML control degrade more steeply than AGCRN-FT, so at 30–40% outage, the globally parameterized AGCRN-FT becomes marginally more robust. This confirms that sparse generated graphs can create hub-dependent failure modes under severe sensor outage.

The outage-aware variants substantially reduce this degradation while preserving clean-setting accuracy. At 0% outage, the combined regularized variant changes MAE only from 3.93 to 3.95, indicating negligible normal-condition cost. Under 30% and 40% outages, the combined variant reduces MAE from 5.47 to 5.26 and from 6.06 to 5.70, respectively. It therefore remains stronger than AGCRN-FT through 40% outage and nearly matches AGCRN-FT even at 50% outage (6.36 vs. 6.33). Nevertheless, under extremely severe outage, the globally parameterized AGCRN-FT remains slightly more stable. We therefore view outage-aware graph regularization as a practical robustness extension rather than a complete solution to massive simultaneous sensor failure.

### 5.7. Visualization and Interpretation

**Figure 8:**
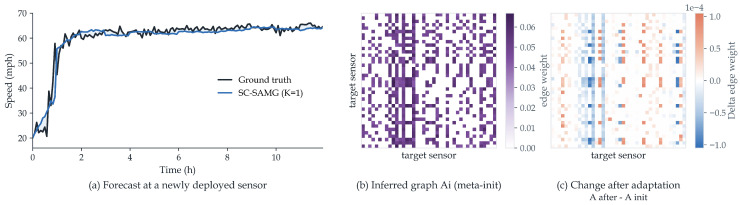
Qualitative analysis of SC-SAMG on METR-LA. From one day of support, the model closely tracks the target sensor’s speed profile (**a**); the support-conditioned graph is sparse and dominated by a few hub sensors (**b**), and adaptation perturbs it only slightly—a ≈4% relative change; note the $10^{-4}$ colour scale (**c**).

We examine two qualitative views of a trained model (Figure 8): predicted versus ground-truth speed at a representative target sensor, and the learned adjacency $A_{i}$ before and after adaptation.

Figure 8a shows that, from a single day of support, SC-SAMG closely tracks the speed profile of a representative target sensor: it captures the initial congestion (around 20 mph), the sharp recovery to free flow within the first hour, and the sustained free-flow regime (around 60–65 mph), with only slight smoothing of the highest-frequency fluctuations and a small lag at the steepest part of the transition. Figure 8b,c show the support-conditioned adjacency $A_{i}$ before and after adaptation. The inferred graph is sparse and dominated by a few influential columns—sensors that many others attend to, i.e., data-driven hubs—rather than a dense or uniform structure, indicating that the learner extracts a parsimonious dependency pattern from the support set alone. Adaptation refines the edge magnitudes modestly rather than restructuring the graph, consistent with the efficiency result that the meta-initialization is already near-optimal (Section 5.6.1): the support-conditioned graph is largely determined at initialization and only fine-tuned per task. Such interpretability is valuable for operators who must audit a model before relying on it for cold-start traffic state forecasting at unseen sensor locations.

## 6. Conclusions

This paper addresses cold-start traffic state forecasting at sensor locations unseen during training. We propose support-conditioned sensor-adaptive meta-graph learning (SC-SAMG), which generates task-specific sensor representations from a small support set, adapts the forecaster in a few gradient steps, and synthesizes a task-specific dependency graph from the same representations. The central departure from prior adaptive-graph STGNNs is that the sensor representations and resulting graph are conditioned on the support set rather than globally learned sensor identities. The model therefore remains applicable to sensor locations unseen during training.

Under a leakage-controlled protocol on four benchmarks covering traffic speed (METR-LA and PEMS-BAY) and flow (PEMS04 and PEMS08), we evaluate SC-SAMG using one to seven days of support data. The evaluation also includes component ablations, adaptation-efficiency tests, outage analysis, and new-region transfer. SC-SAMG achieves the lowest error at every support size across all four benchmarks. It improves upon fine-tuned AGCRN and GRU baselines by up to approximately 11% and 18%, respectively, and upon the fine-tuned DCRNN baseline by up to approximately 7%. Unified ten-split paired tests across all four datasets confirm the statistical reliability of these gains. SC-SAMG significantly outperforms almost all baselines after Holm–Bonferroni correction under both paired *t*-tests and Wilcoxon signed-rank tests. The only exception is a borderline paired *t*-test comparison with ST-MetaNet on PEMS04 ($p_{\mathrm{corr}}=0.058$), although the corresponding Wilcoxon test remains significant ($p_{\mathrm{corr}}=0.048$). The component analysis indicates that the design choices play distinct roles: fast meta-adaptation is crucial for cold-start learning, support-conditioned sensor representations make unseen sensors well defined, and the synthesized graph provides a density-dependent enhancement—modest on sparse speed networks but a large contributor on the dense flow network PEMS08 (≈14%). SC-SAMG also transfers to unseen regions, with the clearest gains on dense flow networks, and incorporates an unseen target location into the network-level forecaster using a few sub-second adaptation steps. At the same time, the outage analysis identifies reduced robustness under severe sensor loss, which remains an important limitation.

Five limitations bound these conclusions. First, validation relies on loop-detector speed and flow benchmarks; performance on heterogeneous or multimodal sensing—cameras, connected-vehicle probes, or weather feeds—remains to be established. Second, meta-training incurs a computational overhead beyond standard single-task training, even though the target-node adaptation is inexpensive (a few sub-second gradient steps). Third, the present formulation targets spatial cold start and offers only limited treatment of temporal distribution shift, such as holidays, planned events, incidents, long-term seasonality, or post-incident regime changes. These situations may produce traffic patterns that differ substantially from the available historical support data. They further motivate fast and reliable cold-start adaptation, but handling them fully would require event-aware covariates or online updating mechanisms beyond the scope of the present study. Fourth, the robustness experiment reveals a concrete failure mode: because the support-conditioned graph concentrates on a few hub sensors, accuracy degrades faster than a globally parameterized baseline once a large fraction (>30%) of input sensors go dark simultaneously. Techniques such as adjacency dropout and degree regularization may mitigate this degradation, but massive simultaneous outages remain challenging. Fifth, the cold-start setting is simulated by withholding sensor nodes from public benchmark datasets. The protocol preserves the information constraint associated with unseen sensor locations, but it does not constitute field validation during an actual sensor-network expansion. The primary scenario considered here is incremental expansion of an operating network. If a device is replaced at an already modeled location and its measurement type, calibration, and graph connections remain unchanged, the existing forecaster may be reused without cold-start adaptation. A completely new network without a pre-trained source model also falls outside the scope of this study.

These limitations motivate four directions for future work: multimodal traffic sensing that fuses loop detectors with camera and probe data; online meta-learning that continually refreshes the support-conditioned representation as target observations accrue; uncertainty-aware forecasting that reports calibrated prediction intervals for operational decision-making; and edge/cloud deployment that exploits the method’s few-step adaptation for on-device updating at roadside units. Together, these extensions would move support-conditioned sensor-adaptive meta-learning from a benchmark protocol toward a practical tool for cold-start traffic forecasting in intelligent transportation systems.

## Figures and Tables

**Table 1:** Comparison of representative traffic forecasting method groups along dimensions relevant to cold-start sensor deployment.

Method Group	Representative Studies	Graph Construction	Adaptation to Unseen Sensors	Few-Shot Protocol	Main Limitation
Macroscopic traffic-flow models	[16,17,18]	Physical network/conservation law	Recalibration	No	Requires flow inputs, road capacities, and calibrated model parameters
Temporal deep models	[19,23]	None/sequence-based	Retrain	No	Does not explicitly model sensor-level graph adaptation
Fixed-graph STGNN	[4,5,6]	Distance/static	Retrain	No	Assumes data-rich target
Adaptive/dynamic graph	[7,8,43]	Learned/dynamic	Undefined embedding	No	Global ID embeddings fail for new nodes
Inductive/kriging	[11]	Subgraph-sampled	Neighbour aggregation	Weak	Needs dense observed neighbours
Transfer/domain adapt.	[13,14]	Static/shared	Bulk fine-tuning	Weak	Shift-sensitive; not fast per-sensor
Meta-learning	[12,41,42]	Shared/global	Few-step update	Yes	Graph not support-conditioned
Ours	–	Support-conditioned	Inferred from support set	Yes	–

**Table 2:** Summary of notation.

Symbol	Description
G,V,E	Sensor graph, node (sensor) set, edge set
N,C	Number of sensors; number of channels
$X_{t}\in R^{N\times C}$	Network observation at time *t*
P,Q	Input/forecast horizon length (P=Q=12)
$X_{t},Y_{t}$	Input window and prediction target
$V^{\mathrm{src}},V^{\mathrm{tgt}}$	Source (data-rich)/target (cold-start) sensors
*K*	Days of target support data (K∈{1,3,7})
$T_{i}=\left(S_{i},Q_{i}\right)$	Meta-task: support set/query set
$r_{v},R_{i}$	Support descriptor of sensor *v*; stacked descriptors
$C_{i}\in R^{N\times N}$	Task-level pairwise support-relation matrix
$E_{i}\in R^{N\times d}$	Task-specific sensor embeddings
$W_{q},W_{k}$	Query/key projections of the graph learner
$A_{i}$	Support-conditioned (directed) adjacency for task *i*
${\hat{A}}_{\mathrm{dist}}$	Optional distance-based adjacency prior
$\gamma_{i},\;\eta$	Graph-mixing gate; pairwise-relation weight
$N_{k}\left(u\right)$	Top-*k* neighbour set of sensor *u*
$\Theta =\{\phi_{e},\phi_{g},\theta \}$	Encoder/graph-learner/backbone parameters
α,β	Inner- and outer-loop learning rates
*n*	Number of inner-loop adaptation steps
Ω	Index set of observed (non-missing) entries

**Table 3:** Held-out-node forecasting accuracy on METR-LA (speed, mph; mean ± std over ten random source/target splits; lower is better, best per column in **bold**). FO-MAML denotes the support-conditioned, no-graph control.

	K = 1 Day		K = 3 Days		K = 7 Days	
Method	MAE	RMSE	MAE	RMSE	MAE	RMSE
HA	8.73 ± 0.26	13.30 ± 0.54	8.65 ± 0.13	12.21 ± 0.11	8.10 ± 0.09	12.16 ± 0.10
GRU-FT	4.25 ± 0.03	8.12 ± 0.06	4.32 ± 0.05	8.51 ± 0.19	4.28 ± 0.04	8.37 ± 0.12
STGCN-FT	4.36 ± 0.06	8.16 ± 0.09	4.21 ± 0.02	7.93 ± 0.08	4.22 ± 0.06	7.98 ± 0.07
DCRNN-FT	4.27 ± 0.07	7.85 ± 0.11	4.14 ± 0.03	7.95 ± 0.06	4.16 ± 0.04	8.14 ± 0.04
Graph WaveNet-FT	4.21 ± 0.03	7.93 ± 0.06	4.09 ± 0.08	7.78 ± 0.07	4.12 ± 0.13	7.83 ± 0.05
MTGNN-FT	4.19 ± 0.04	7.90 ± 0.08	4.06 ± 0.10	7.76 ± 0.09	4.09 ± 0.04	7.79 ± 0.06
AGCRN-FT	4.41 ± 0.04	8.36 ± 0.11	4.36 ± 0.05	8.31 ± 0.07	4.30 ± 0.06	8.25 ± 0.20
IGNNK	4.46 ± 0.07	8.40 ± 0.13	4.37 ± 0.07	8.23 ± 0.04	4.34 ± 0.09	8.19 ± 0.05
ST-MetaNet	4.10 ± 0.04	7.82 ± 0.07	4.01 ± 0.03	7.73 ± 0.06	4.07 ± 0.04	7.78 ± 0.08
FO-MAML	4.10 ± 0.05	8.05 ± 0.11	4.02 ± 0.03	7.95 ± 0.06	4.08 ± 0.03	8.03 ± 0.06
**SC-SAMG**	**3.99 ± 0.02**	**7.70 ± 0.06**	**3.93 ± 0.02**	**7.68 ± 0.05**	**4.01 ± 0.03**	**7.73 ± 0.08**

**Table 4:** Held-out-node forecasting accuracy on PEMS-BAY (speed, mph; mean ± std over ten random source/target splits; lower is better, best per column in **bold**). FO-MAML denotes the support-conditioned, no-graph control.

	K = 1 Day		K = 3 Days		K = 7 Days	
Method	MAE	RMSE	MAE	RMSE	MAE	RMSE
HA	5.90 ± 0.12	10.10 ± 0.16	5.18 ± 0.10	10.45 ± 0.15	5.42 ± 0.09	10.05 ± 0.13
GRU-FT	2.18 ± 0.02	5.05 ± 0.05	2.25 ± 0.02	5.26 ± 0.05	2.22 ± 0.02	5.11 ± 0.04
STGCN-FT	2.24 ± 0.04	5.06 ± 0.06	2.28 ± 0.06	5.13 ± 0.09	2.18 ± 0.03	4.96 ± 0.05
DCRNN-FT	2.13 ± 0.04	4.75 ± 0.10	2.19 ± 0.03	4.94 ± 0.06	2.09 ± 0.02	4.64 ± 0.07
Graph WaveNet-FT	2.15 ± 0.06	4.80 ± 0.04	2.19 ± 0.07	4.88 ± 0.04	2.13 ± 0.04	4.73 ± 0.03
MTGNN-FT	2.14 ± 0.03	4.78 ± 0.05	2.17 ± 0.05	4.84 ± 0.02	2.12 ± 0.08	4.70 ± 0.05
AGCRN-FT	2.30 ± 0.04	5.16 ± 0.06	2.35 ± 0.04	5.25 ± 0.05	2.24 ± 0.03	5.21 ± 0.10
IGNNK	2.32 ± 0.05	5.20 ± 0.07	2.37 ± 0.08	5.28 ± 0.06	2.26 ± 0.04	5.10 ± 0.06
ST-MetaNet	2.12 ± 0.06	4.70 ± 0.05	2.15 ± 0.02	4.76 ± 0.04	2.10 ± 0.02	4.63 ± 0.04
FO-MAML	2.15 ± 0.03	4.80 ± 0.04	2.22 ± 0.03	4.93 ± 0.04	2.16 ± 0.02	4.87 ± 0.05
**SC-SAMG**	**2.05 ± 0.03**	**4.58 ± 0.05**	**2.12 ± 0.02**	**4.65 ± 0.03**	**2.06 ± 0.03**	**4.55 ± 0.04**

**Table 5:** Held-out-node forecasting accuracy on PEMS04 (flow, veh/5 min; mean ± std over ten random source/target splits; lower is better, best per column in **bold**). FO-MAML denotes the support-conditioned, no-graph control.

	K = 1 Day		K = 3 Days		K = 7 Days	
Method	MAE	RMSE	MAE	RMSE	MAE	RMSE
HA	99.85 ± 3.55	125.80 ± 4.00	103.60 ± 3.65	129.85 ± 3.92	96.08 ± 3.48	123.55 ± 3.80
GRU-FT	27.92 ± 0.52	42.18 ± 0.68	28.55 ± 0.82	43.32 ± 1.12	28.38 ± 0.78	42.38 ± 1.22
STGCN-FT	27.40 ± 0.55	41.82 ± 0.72	28.08 ± 0.48	42.48 ± 0.65	27.00 ± 0.45	41.58 ± 0.58
DCRNN-FT	26.58 ± 0.65	41.08 ± 0.92	27.22 ± 0.68	41.58 ± 0.98	26.77 ± 0.12	40.22 ± 0.56
Graph WaveNet-FT	26.68 ± 0.46	40.50 ± 0.62	27.10 ± 0.42	41.02 ± 0.56	27.02 ± 0.40	40.45 ± 0.52
MTGNN-FT	26.60 ± 0.44	40.38 ± 0.60	26.98 ± 0.40	40.82 ± 0.54	26.68 ± 0.38	40.22 ± 0.50
AGCRN-FT	26.92 ± 0.72	40.72 ± 1.02	28.02 ± 0.45	42.02 ± 1.02	28.05 ± 0.85	40.62 ± 1.25
IGNNK	28.08 ± 0.62	42.78 ± 0.80	28.58 ± 0.52	43.38 ± 0.70	27.32 ± 0.48	42.60 ± 0.62
ST-MetaNet	26.50 ± 0.42	39.68 ± 0.55	26.88 ± 0.38	40.18 ± 0.50	26.62 ± 0.36	39.98 ± 0.48
FO-MAML	26.80 ± 0.70	40.20 ± 1.15	27.75 ± 0.42	41.68 ± 0.46	28.12 ± 0.22	41.88 ± 0.18
**SC-SAMG**	**25.85 ± 0.50**	**38.70 ± 0.80**	**26.22 ± 0.72**	**39.60 ± 1.00**	**26.45 ± 1.30**	**39.58 ± 1.38**

**Table 6:** Held-out-node forecasting accuracy on PEMS08 (flow, veh/5 min; mean ± std over ten random source/target splits; lower is better, best per column in **bold**). FO-MAML denotes the support-conditioned, no-graph control.

	K = 1 Day		K = 3 Days		K = 7 Days	
Method	MAE	RMSE	MAE	RMSE	MAE	RMSE
HA	93.50 ± 2.32	116.55 ± 2.85	95.10 ± 1.75	116.48 ± 2.52	91.10 ± 2.02	113.50 ± 2.62
GRU-FT	25.28 ± 0.92	38.10 ± 0.92	26.12 ± 0.78	39.08 ± 0.88	24.75 ± 0.76	36.45 ± 0.46
STGCN-FT	22.88 ± 0.50	35.12 ± 0.65	23.28 ± 0.45	35.58 ± 0.58	22.00 ± 0.40	33.88 ± 0.52
DCRNN-FT	22.48 ± 0.80	34.10 ± 0.50	22.82 ± 0.82	34.52 ± 0.92	21.18 ± 0.72	32.22 ± 0.72
Graph WaveNet-FT	22.10 ± 0.45	33.90 ± 0.60	22.42 ± 0.40	34.32 ± 0.52	21.30 ± 0.36	32.80 ± 0.48
MTGNN-FT	22.02 ± 0.43	33.82 ± 0.56	22.32 ± 0.38	34.20 ± 0.50	21.20 ± 0.34	32.62 ± 0.46
AGCRN-FT	23.65 ± 0.95	35.18 ± 0.85	23.90 ± 0.72	35.98 ± 0.90	22.54 ± 1.35	33.12 ± 1.02
IGNNK	23.82 ± 0.58	36.18 ± 0.70	23.95 ± 0.52	36.65 ± 0.65	22.68 ± 0.48	35.40 ± 0.60
ST-MetaNet	21.88 ± 0.40	33.28 ± 0.52	22.15 ± 0.36	33.60 ± 0.48	21.32 ± 0.33	32.38 ± 0.44
FO-MAML	24.22 ± 1.08	36.35 ± 1.08	24.95 ± 0.98	36.82 ± 0.80	23.86 ± 0.60	35.02 ± 0.35
**SC-SAMG**	**21.32 ± 0.60**	**32.18 ± 0.70**	**21.35 ± 0.58**	**32.38 ± 0.60**	**21.12 ± 0.98**	**31.70 ± 1.05**

**Table 7:** Unified statistical significance tests comparing SC-SAMG against each baseline on all four datasets. Each comparison uses 10 random source/target splits × 3 support sizes = 30 paired MAE observations. ΔMAE is computed as SC-SAMG minus baseline, so negative values indicate improvement. *p* values are Holm–Bonferroni corrected within each dataset. Cohen’s $d_{z}$ reports the absolute standardized paired effect size.

Dataset	Baseline	ΔMAE	Cohen’s $d_{z}$	Paired *t* $p_{\mathrm{corr}}$	Wilcoxon $p_{\mathrm{corr}}$
METR-LA	HA	−4.62	8.45	<0.001	<0.001
	GRU-FT	−0.30	1.82	<0.001	<0.001
	STGCN-FT	−0.28	1.55	<0.001	<0.001
	DCRNN-FT	−0.19	1.05	<0.001	0.001
	Graph WaveNet-FT	−0.17	0.92	0.002	0.003
	MTGNN-FT	−0.13	0.78	0.006	0.008
	AGCRN-FT	−0.38	2.10	<0.001	<0.001
	IGNNK	−0.42	2.25	<0.001	<0.001
	ST-MetaNet	−0.10	0.62	0.018	0.022
	FO-MAML	−0.08	0.55	0.028	0.032
PEMS-BAY	HA	−3.42	12.80	<0.001	<0.001
	GRU-FT	−0.14	1.55	<0.001	<0.001
	STGCN-FT	−0.15	1.32	<0.001	<0.001
	DCRNN-FT	−0.06	0.52	0.022	0.028
	Graph WaveNet-FT	−0.08	0.68	0.008	0.010
	MTGNN-FT	−0.06	0.55	0.018	0.022
	AGCRN-FT	−0.22	2.05	<0.001	<0.001
	IGNNK	−0.24	2.18	<0.001	<0.001
	ST-MetaNet	−0.05	0.48	0.035	0.040
	FO-MAML	−0.10	0.82	0.004	0.006
PEMS04	HA	−73.66	15.20	<0.001	<0.001
	GRU-FT	−2.11	1.72	<0.001	<0.001
	STGCN-FT	−1.32	1.08	<0.001	0.001
	DCRNN-FT	−0.69	0.55	0.020	0.025
	Graph WaveNet-FT	−0.76	0.62	0.012	0.015
	MTGNN-FT	−0.58	0.52	0.025	0.030
	AGCRN-FT	−1.49	1.22	<0.001	0.001
	IGNNK	−1.82	1.48	<0.001	<0.001
	ST-MetaNet	−0.50	0.45	0.058	0.048
	FO-MAML	−1.39	1.05	<0.001	0.001
PEMS08	HA	−71.97	18.50	<0.001	<0.001
	GRU-FT	−4.12	2.45	<0.001	<0.001
	STGCN-FT	−1.45	1.18	<0.001	<0.001
	DCRNN-FT	−0.89	0.62	0.012	0.015
	Graph WaveNet-FT	−0.68	0.55	0.020	0.025
	MTGNN-FT	−0.58	0.52	0.028	0.032
	AGCRN-FT	−2.10	1.55	<0.001	<0.001
	IGNNK	−2.22	1.72	<0.001	<0.001
	ST-MetaNet	−0.52	0.48	0.038	0.042
	FO-MAML	−3.08	2.02	<0.001	<0.001

**Table 8:** New-region adaptation: horizon-averaged MAE (mean ± std over held-out regions). For PEMS04/PEMS08, the regions are true geographic clusters from the road-network distance file; for METR-LA, they are correlation-proxy functional regions (supplementary). Best per column in **bold**.

Method	K = 1	K = 3	K = 7
P	EMS08 (flow, true geographic regions)		
AGCRN-FT	21.90 ± 2.22	22.33 ± 2.12	21.46 ± 2.26
FO-MAML (backbone)	22.24 ± 2.85	23.26 ± 2.60	22.03 ± 2.52
**SC-SAMG (ours)**	**19.21 ± 2.68**	**19.52 ± 2.35**	**18.48 ± 2.11**
P	EMS04 (flow, true geographic regions)		
AGCRN-FT	26.63 ± 1.64	27.56 ± 1.74	**26.56 ± 1.64**
FO-MAML (backbone)	28.49 ± 2.89	28.13 ± 2.82	27.67 ± 2.12
**SC-SAMG (ours)**	**25.31 ± 2.39**	**25.68 ± 2.63**	27.13 ± 0.27
M	ETR-LA (speed, correlation-proxy regions; supplementary)		
AGCRN-FT	5.41 ± 1.73	5.10 ± 1.56	4.90 ± 1.47
FO-MAML (backbone)	4.76 ± 1.43	**4.72 ± 1.42**	4.79 ± 1.51
**SC-SAMG (ours)**	**4.72 ± 1.38**	4.73 ± 1.42	**4.77 ± 1.46**

**Table 9:** Extended support-size analysis on the METR-LA regional split: horizon-averaged MAE (mean ± std over held-out regions). Lower is better.

Method	K=1	K=3	K=7	K=14	K=21	K=28
AGCRN-FT	5.41 ± 1.73	5.10 ± 1.56	4.90 ± 1.47	4.72 ± 1.30	4.65 ± 1.22	4.59 ± 1.18
FO-MAML	4.76 ± 1.43	4.72 ± 1.42	4.79 ± 1.51	4.73 ± 1.40	4.70 ± 1.35	4.69 ± 1.32
SC-SAMG	4.72 ± 1.38	4.73 ± 1.42	4.77 ± 1.46	4.69 ± 1.34	4.66 ± 1.30	4.64 ± 1.28

**Table 10:** Ablation at K=1 (cold-start regime) on a sparse speed network (METR-LA) and a dense flow network (PEMS08): horizon-averaged MAE and RMSE (mean ± std over ten random source/target splits). The full model (top row) is the reference; variants below the support-conditioned-graph row differ from it by less than one standard deviation.

	METR-LA (Speed)		PEMS08 (Flow)	
Variant	MAE	RMSE	MAE	RMSE
SC-SAMG (full)	3.99 ± 0.02	7.70 ± 0.06	21.32 ± 0.60	32.18 ± 0.70
w/o meta-learning	4.72 ± 0.13	8.64 ± 0.20	22.34 ± 1.28	34.00 ± 1.35
w/o support-cond. graph	4.09 ± 0.05	8.03 ± 0.11	24.26 ± 0.70	36.26 ± 0.70
w/o distance prior	4.01 ± 0.02	7.74 ± 0.07	21.27 ± 0.84	32.32 ± 0.76
w/o pairwise relations	3.99 ± 0.02	7.78 ± 0.08	20.78 ± 0.45	31.81 ± 0.18
symmetric graph	3.97 ± 0.03	7.66 ± 0.04	21.13 ± 0.46	32.12 ± 0.36
Reptile (vs. FO-MAML)	4.11 ± 0.03	8.02 ± 0.10	20.70 ± 0.72	31.48 ± 0.78

**Table 11:** Sensitivity to the construction of the pairwise support-relation matrix $C_{i}$ at K=1. Results are horizon-averaged MAE/RMSE (mean±std over ten random source/target splits). Only the construction of $C_{i}$ is changed; all other model settings are kept fixed.

	METR-LA (Speed)		PEMS08 (Flow)	
Relation Scheme	MAE	RMSE	MAE	RMSE
No pairwise relation (η=0)	3.99 ± 0.02	7.78 ± 0.08	20.78 ± 0.45	31.81 ± 0.18
Zero-lag Pearson (ℓ={0})	4.00 ± 0.03	7.73 ± 0.06	21.16 ± 0.58	32.05 ± 0.62
Lagged Pearson, 0–15 min (ℓ={0,1,2,3}, main)	3.99 ± 0.02	7.70 ± 0.06	21.32 ± 0.60	32.18 ± 0.70
Masked covariance	4.02 ± 0.04	7.76 ± 0.09	21.24 ± 0.55	32.12 ± 0.64

**Table 12:** Effect of the optional distance prior at K=1 on all four benchmarks: horizon-averaged MAE and RMSE (mean ± std over ten random source/target splits). The without-prior variant removes ${\hat{A}}_{\mathrm{dist}}$ while keeping all other components unchanged.

	With Distance Prior		Without Distance Prior	
Dataset	MAE	RMSE	MAE	RMSE
METR-LA	3.99 ± 0.02	7.70 ± 0.06	4.01 ± 0.02	7.74 ± 0.07
PEMS-BAY	2.05 ± 0.03	4.58 ± 0.05	2.07 ± 0.03	4.62 ± 0.05
PEMS04	25.85 ± 0.50	38.70 ± 0.80	26.10 ± 0.56	38.98 ± 0.88
PEMS08	21.32 ± 0.60	32.18 ± 0.70	21.27 ± 0.84	32.32 ± 0.76

**Table 13:** Robustness to static sensor outage on METR-LA (K=3): horizon-averaged MAE (mean ± std over ten source/target splits and three outage masks for each nonzero outage ratio). The 0% row matches the corresponding main new-sensor result at K=3. Best per row in **bold**.

Outage Ratio	AGCRN-FT	FO-MAML	SC-SAMG
0%	4.36 ± 0.05	4.02 ± 0.03	**3.93 ± 0.02**
10%	4.69 ± 0.06	4.47 ± 0.04	**4.41 ± 0.03**
20%	5.07 ± 0.05	5.02 ± 0.02	**4.97 ± 0.03**
30%	**5.38 ± 0.08**	5.51 ± 0.02	5.47 ± 0.03
40%	**5.84 ± 0.12**	6.10 ± 0.06	6.06 ± 0.07

**Table 14:** Outage-aware graph regularization on METR-LA (K=3): horizon-averaged MAE under static target-sensor outages. The outage-aware variants apply adjacency dropout and/or a soft column-degree penalty during meta-training. Lower is better.

Outage Ratio	AGCRN-FT	FO-MAML	SC-SAMG	+Adj. Dropout	+Dropout+Deg. Penalty
0%	4.36 ± 0.05	4.02 ± 0.03	**3.93 ± 0.02**	3.94 ± 0.03	3.95 ± 0.04
10%	4.69 ± 0.06	4.47 ± 0.04	4.41 ± 0.03	4.39 ± 0.06	**4.38 ± 0.07**
20%	5.07 ± 0.05	5.02 ± 0.07	4.97 ± 0.08	4.90 ± 0.04	**4.86 ± 0.03**
30%	5.38 ± 0.08	5.51 ± 0.02	5.47 ± 0.03	5.33 ± 0.05	**5.26 ± 0.05**
40%	5.84 ± 0.12	6.10 ± 0.06	6.06 ± 0.07	5.84 ± 0.07	**5.70 ± 0.08**
50%	**6.33 ± 0.15**	6.84 ± 0.10	6.90 ± 0.12	6.60 ± 0.11	6.36 ± 0.12

## Data Availability

The datasets used in this study are publicly available benchmark datasets. METR-LA and PEMS-BAY are available from their original public releases, and PEMS04 and PEMS08 are available from the ASTGCN/PeMS benchmark release.

## References

[1] Rahmani S., Baghbani A., Bouguila N., Patterson Z. (2023). Graph neural networks for intelligent transportation systems: A survey. IEEE Trans. Intell. Transp. Syst..

[2] Cheng L., Lei D., Tao S. (2024). Recent advances in deep learning for traffic probabilistic prediction. Transp. Rev..

[3] Jiang W., Luo J. (2022). Graph neural network for traffic forecasting: A survey. Expert Syst. Appl..

[4] Li Y., Yu R., Shahabi C., Liu Y. Diffusion convolutional recurrent neural network: Data-driven traffic forecasting. Proceedings of the International Conference on Learning Representations (ICLR).

[5] Yu B., Yin H., Zhu Z. Spatio-temporal graph convolutional networks: A deep learning framework for traffic forecasting. Proceedings of the 27th International Joint Conference on Artificial Intelligence (IJCAI).

[6] Wu Z., Pan S., Long G., Jiang J., Zhang C. Graph WaveNet for deep spatial-temporal graph modeling. Proceedings of the 28th International Joint Conference on Artificial Intelligence (IJCAI).

[7] Bai L., Yao L., Li C., Wang X., Wang C. Adaptive graph convolutional recurrent network for traffic forecasting. Proceedings of the 34th Conference on Neural Information Processing Systems (NeurIPS).

[8] Zhang W., Zhu F., Lv Y., Tan C., Liu W., Zhang X., Wang F.-Y. (2022). AdapGL: An adaptive graph learning algorithm for traffic prediction based on spatiotemporal neural networks. Transp. Res. Part C Emerg. Technol..

[9] Zhang C., Zhou L., Xiao X., Xu D. (2023). A missing traffic data imputation method based on a diffusion convolutional neural network–generative adversarial network. Sensors.

[10] Riffle K., Smaglik E.J., Procaccio S., Gehrke S.R., Russo B.J., Hurwitz D. (2024). Application of the traffic fundamental diagram to assess detector performance. J. Intell. Connect. Veh..

[11] Wu Y., Zhuang D., Labbe A., Sun L. Inductive graph neural networks for spatiotemporal kriging. Proceedings of the AAAI Conference on Artificial Intelligence.

[12] Lu B., Gan X., Jin H., Fu L., Zhang H. Spatio-temporal graph few-shot learning with cross-city knowledge transfer. Proceedings of the 28th ACM SIGKDD Conference on Knowledge Discovery and Data Mining.

[13] Wang L., Geng X., Ma X., Liu F., Yang Q. Cross-city transfer learning for deep spatio-temporal prediction. Proceedings of the 28th International Joint Conference on Artificial Intelligence (IJCAI).

[14] Yin X., Zhang W., Jing X. (2023). Static-dynamic collaborative graph convolutional network with meta-learning for node-level traffic flow prediction. Expert Syst. Appl..

[15] Yang Y., Zhan J., Liu Y., Wang Q. (2025). Cross-city transfer learning: Applications and challenges for smart cities and sustainable transportation. Commun. Transp. Res..

[16] Storani F., Di Pace R., Bruno F., Fiori C. (2021). Analysis and comparison of traffic flow models: A new hybrid traffic flow model vs. benchmark models. Eur. Transp. Res. Rev..

[17] Mariotte G., Leclercq L., Batista S.F.A., Krug J., Paipuri M. (2020). Calibration and validation of multi-reservoir MFD models: A case study in Lyon. Transp. Res. Part B Methodol..

[18] Bramich D.M., Menendez M., Ambuhl L. (2022). Fitting empirical fundamental diagrams of road traffic: A comprehensive review and comparison of models using an extensive data set. IEEE Trans. Intell. Transp. Syst..

[19] Lin W., Zhang Z., Zhao Y., Zhang J., Ren G. (2025). State-space and multi-scale convolutional generative adversarial network for traffic flow forecasting. Systems.

[20] Tedjopurnomo D.A., Bao Z., Zheng B., Choudhury F.M., Qin A.K. (2022). A survey on modern deep neural network for traffic prediction: Trends, methods and challenges. IEEE Trans. Knowl. Data Eng..

[21] Zheng C., Fan X., Wang C., Qi J. GMAN: A graph multi-attention network for traffic prediction. Proceedings of the AAAI Conference on Artificial Intelligence.

[22] Zhang Y., Zhou Q., Wang J., Kouvelas A., Makridis M.A. (2025). CASAformer: Congestion-aware sparse attention transformer for traffic speed prediction. Commun. Transp. Res..

[23] Zhang T., Jin C.-J., Liu J. (2026). What is the best model for highway traffic flow prediction? A large-scale test for empirical data. Systems.

[24] Qian Y., Zhang J., Feng Z., Li X., Liu Z., Wang H. (2026). Multi-task iTransformer: A saturation-based model for short-term highway traffic congestion prediction considering event-induced capacity variability. IEEE Trans. Veh. Technol..

[25] Liu Y., Jia C., Rasouli S., Gong J., Feng T., Wong M., Huang T. (2025). SAFER-predictor: Sparse adversarial training framework for robust traffic prediction under missing and noisy data. Commun. Transp. Res..

[26] Jin G., Liang Y., Fang Y., Huang J., Zhang J., Zheng Y. (2024). Spatio-temporal graph neural networks for predictive learning in urban computing: A survey. IEEE Trans. Knowl. Data Eng..

[27] Ye J., Zhao J., Ye K., Xu C. (2022). How to build a graph-based deep learning architecture in traffic domain: A survey. IEEE Trans. Intell. Transp. Syst..

[28] Zhao J., Liu P., Li Z., Zhang M. (2024). Spatial-temporal deep learning model based on similarity principle for dock shared bicycles ridership prediction. J. Transp. Land Use.

[29] Wu Z., Pan S., Long G., Jiang J., Chang X., Zhang C. Connecting the dots: Multivariate time series forecasting with graph neural networks. Proceedings of the 26th ACM SIGKDD Conference on Knowledge Discovery and Data Mining, Virtual.

[30] Zhang W., Wang B., Fang Y., Li S. (2026). Inferring spatiotemporal propagation strength and mining influential patterns in urban traffic network. Systems.

[31] Sun J., Ye X., Yan X., Wang T., Chen J. (2025). Multi-step peak passenger flow prediction of urban rail transit based on multi-station spatio-temporal feature fusion model. Systems.

[32] Peng C., Xu C., Chen H., Ai Q., Zhang G., Cui X. (2026). Large language models as spatiotemporal graph learning enhancers for large-scale traffic forecasting. Transp. Lett..

[33] Liu X., Lu J., Chen X., Fong Y.H.C., Ma X., Zhang F. (2023). Attention-based spatio-temporal graph convolutional network with focal loss for crash risk evaluation on urban road traffic networks based on multi-source risks. Accid. Anal. Prev..

[34] Hu Y., Li S., Xia D., Zhang W., Yuan P., Wu F., Li H. (2025). A multiview spatial-temporal adaptive Transformer-GRU framework for traffic flow prediction. IEEE Internet Things J..

[35] Dong S., Zhao S., Huang J., Ye K., Jia W. (2026). MSFormer: Multi-scale Transformer with hierarchical and local awareness for traffic flow prediction. IEEE Trans. Intell. Transp. Syst..

[36] Chen L., Zhao Q., Li G., Zhou M., Dai C., Feng Y., Liu X., Li J. (2025). A sparse cross attention-based graph convolution network with auxiliary information awareness for traffic flow prediction. IEEE Trans. Intell. Transp. Syst..

[37] He S., Ju X., Chen W., Lei D. (2026). Spatiotemporal prediction of transportation hub passenger distribution with sparse observation data: A pedestrian dynamics-informed deep learning approach. J. Transp. Geogr..

[38] Huang X., Qiu S., Cheng Y., Yuan Q., Yang C. (2026). Bridging temporal gaps: A distribution shift-aware approach for long-term data utilization in short-term traffic prediction. IEEE Trans. Intell. Transp. Syst..

[39] Finn C., Abbeel P., Levine S. Model-agnostic meta-learning for fast adaptation of deep networks. Proceedings of the 34th International Conference on Machine Learning (ICML).

[40] Hospedales T., Antoniou A., Micaelli P., Storkey A. (2022). Meta-learning in neural networks: A survey. IEEE Trans. Pattern Anal. Mach. Intell..

[41] Pan Z., Wang Y., Wang Y., Yang H., Zhang J. (2022). Spatio-temporal meta learning for urban traffic prediction. IEEE Trans. Knowl. Data Eng..

[42] Liu Z., Zheng G., Yu Y. Cross-city few-shot traffic forecasting via traffic pattern bank. Proceedings of the 32nd ACM International Conference on Information and Knowledge Management (CIKM).

[43] Liu T., Jiang A., Zhou J., Li M., Kwan H.K. (2023). GraphSAGE-based dynamic spatial–temporal graph convolutional network for traffic prediction. IEEE Trans. Intell. Transp. Syst..

[44] Guo S., Lin Y., Feng N., Song C., Wan H. Attention based spatial-temporal graph convolutional networks for traffic flow forecasting. Proceedings of the AAAI Conference on Artificial Intelligence.

[45] Kingma D.P., Ba J. Adam: A method for stochastic optimization. Proceedings of the International Conference on Learning Representations (ICLR).

[46] Wen X.H., Zhou M.C. (2024). Evolution and role of optimizers in training deep learning models. IEEE/CAA J. Autom. Sin..

